# The scaffold protein p62 regulates adaptive thermogenesis through ATF2 nuclear target activation

**DOI:** 10.1038/s41467-020-16230-8

**Published:** 2020-05-08

**Authors:** Katrin Fischer, Anna Fenzl, Dianxin Liu, Kenneth A. Dyar, Maximilian Kleinert, Markus Brielmeier, Christoffer Clemmensen, Anna Fedl, Brian Finan, Andre Gessner, Martin Jastroch, Jianfeng Huang, Susanne Keipert, Martin Klingenspor, Jens C. Brüning, Manfred Kneilling, Florian C. Maier, Ahmed E. Othman, Bernd J. Pichler, Ines Pramme-Steinwachs, Stephan Sachs, Angelika Scheideler, Wolfgang M. Thaiss, Henriette Uhlenhaut, Siegfried Ussar, Stephen C. Woods, Julia Zorn, Kerstin Stemmer, Sheila Collins, Maria Diaz-Meco, Jorge Moscat, Matthias H. Tschöp, Timo D. Müller

**Affiliations:** 1 Institute for Diabetes and Obesity, Helmholtz Diabetes Center (HDC), Helmholtz Zentrum München and German National Diabetes Center (DZD), 85764 Neuherberg, Germany; 2 Division of Cardiovascular Medicine, Vanderbilt University Medical Center, Nashville, TN 37232 USA; 3 Division of Metabolic Diseases, Department of Medicine, Technische Universität München, 80333 Munich, Germany; 4 Section of Molecular Physiology, Department of Nutrition, Exercise and Sports, Faculty of Science, University of Copenhagen, 2200 Copenhagen, Denmark; 5 Research Unit Comparative Medicine, Helmholtz Zentrum München, German Research Center for Environmental Health GmbH, Neuherberg, Germany; 6 Novo Nordisk Foundation Center for Basic Metabolic Research, Faculty of Health and Medical Sciences, University of Copenhagen, Blegdamsvej 3B, 2200 Copenhagen, Denmark; 7 Novo Nordisk Research Center, Indianapolis, IN 46241 USA; 8 Institute of Clinical Microbiology and Hygiene, University Hospital Regensburg, Neuherberg, Germany; 9 Department of Molecular Biosciences, The Wenner-Gren Institute, The Arrhenius Laboratories F3, Stockholm University, SE-106 91 Stockholm, Sweden; 10 Cancer Metabolism and Signaling Networks Program, Sanford Burnham Prebys Medical Discovery Institute, La Jolla, CA 92037 USA; 11 Chair of Molecular Nutritional Medicine, Technical University of Munich, TUM School of Life Sciences Weihenstephan, Gregor-Mendel-Strasse 2, D-85354 Freising, Germany; 12 EKFZ—Else-Kröner Fresenius Center for Nutritional Medicine, Technical University of Munich, Gregor-Mendel-Strasse 2, D-85354 Freising, Germany; 13 Department of Neuronal Control of Metabolism, Max Planck Institute for Metabolism Research, Gleueler Strasse 50, 50931 Cologne, Germany; 14 Policlinic for Endocrinology, Diabetes and Preventive Medicine (PEDP), University Hospital Cologne, Kerpener Strasse 26, 50924 Cologne, Germany; 15 Excellence Cluster on Cellular Stress Responses in Aging-Associated Diseases (CECAD) and Center for Molecular Medicine Cologne (CMMC), University of Cologne, Joseph-Stelzmann-Strasse 26, 50931 Cologne, Germany; 16 Werner Siemens Imaging Center, Department of Preclinical Imaging and Radiopharmacy, Eberhard Karls University Tübingen, Tübingen, Germany; 17 Department of Dermatology, Eberhard Karls University Tübingen, 72076 Tübingen, Germany; 18 Department of Diagnostic and Interventional Radiology, Eberhard Karls University Hospital Tübingen, Hoppe-Seyler-Straße 3, 72076 Tübingen, Germany; 19 Metabolic Disease Institute, Department of Psychiatry and Behavioral Neuroscience, University of Cincinnati, Cincinnati, OH 45237 USA; 20 Department of Biology, University of Konstanz, Konstanz, Germany; 21 Institute of Experimental and Clinical Pharmacology and Pharmacogenomics, Department of Pharmacology, Experimental Therapy and Toxicology, Eberhard Karls University Hospitals and Clinics, 72076 Tübingen, Germany

**Keywords:** Transcriptional regulatory elements, Metabolic disorders, Obesity

## Abstract

During β-adrenergic stimulation of brown adipose tissue (BAT), p38 phosphorylates the activating transcription factor 2 (ATF2) which then translocates to the nucleus to activate the expression of *Ucp1* and *Pgc-1α*. The mechanisms underlying ATF2 target activation are unknown. Here we demonstrate that p62 (Sqstm1) binds to ATF2 to orchestrate activation of the *Ucp1* enhancer and *Pgc-1α* promoter. P62^Δ69-251^ mice show reduced expression of *Ucp1* and *Pgc-1α* with impaired ATF2 genomic binding. Modulation of *Ucp1* and *Pgc-1α* expression through p62 regulation of ATF2 signaling is demonstrated in vitro and in vivo in p62^Δ69-251^ mice, global p62^−/−^ and Ucp1-Cre p62^flx/flx^ mice. BAT dysfunction resulting from p62 deficiency is manifest after birth and obesity subsequently develops despite normal food intake, intestinal nutrient absorption and locomotor activity. In summary, our data identify p62 as a master regulator of BAT function in that it controls the *Ucp1* pathway through regulation of ATF2 genomic binding.

## Abstract (web summary)

Beta-adrenergic stimulation of brown adipose tissue leads to thermogenesis via the activating transcription factor 2 (ATF2) mediated expression of the thermogenic genes *Ucp1* and *Pgc-1α*. Here, the authors show that the scaffold protein p62 regulates brown adipose tissue function through modifying ATF2 genomic binding and subsequent *Ucp1* and *Pgc-1α* induction.

## Introduction

Having long been considered dispensable, brown adipose tissue (BAT) in adult humans is now recognized today as an organ of physiological relevance for energy metabolism^1–3^. In adult humans, the mass and activity of BAT inversely correlates with body mass index (BMI) and percent body fat^4–6^, and sympathetic nervous system (SNS) activation by cold exposure^7–9^, or by β_3_-adrenergic receptor agonism^10^ enhances BAT thermogenesis and energy expenditure. The mitogen-activated protein kinase (MAPK) p38α, the key regulator of adaptive thermogenesis, modulates uncoupling protein 1 (*Ucp1*) and peroxisome proliferator-activated receptor gamma coactivator 1-alpha (*Pgc-1α*) expression via phosphorylation, and action of the activating transcription factor 2 (ATF2)^11,12^. Upon activation by p38α, p-ATF2 translocates to the nucleus and initiates expression of *Ucp1* and *Ppargc1a* (*Pgc-1α*) via binding to cAMP responsive elements (CRE) in the *Ucp1* enhancer and *Pgc-1α* promoter^11^. Highlighting the key role of this pathway in UCP1 action, cold-induced β-adrenergic receptor stimulation fails to promote *Ucp1* expression in mouse BAT primary cells pretreated with the p38α/β MAPK inhibitor SB202190^12^. While nuclear entry and action of ATF2 is crucial for BAT adaptive thermogenesis^11,12^, the mechanism underlying ATF2 target activation is unknown^13^.

Scaffold proteins are key mediators of selective and efficient cell signal transduction, which they achieve through direct and specific interaction with their target proteins. The scaffold protein p62 (sequestosome 1; Sqstm1) is a multimodular adaptor protein involved in key metabolic processes like tissue inflammation, cell differentiation, cell growth, and tumorigenesis^14^. Mice with global^15^ or adipose-specific^16^ deletion of p62 have a severe obese phenotype with normal food intake but decreased energy expenditure and impaired BAT function. Global p62^−/−^ mice also have enhanced adipogenesis with hyperphosphorylation of the extracellular signal-regulated kinase (ERK1/2) in the white adipose tissue (WAT)^15^. Loss of adipogenic capacity by ERK1/2 deletion can prevent obesity in global p62^−/−^ mice^17^, emphasizing uncertainty as to whether the dysregulated energy metabolism in p62-deficient mice originates from enhanced adipogenesis and/or impaired energy expenditure^16^. The aim of this paper was to dissect the molecular foundations underlying energy metabolism control by p62 and to identify the molecular mechanisms of how p62 regulates BAT thermogenesis. Our data demonstrate that mice that lack the amino acids (aa) 69–251 of the p62 protein (p62^Δ69-251^ mice) have normal protein levels of p-ERK1/2 in WAT and show no changes in adipogenesis or adipocyte differentiation. However, these mice develop a severe obese phenotype that is accompanied by impaired energy expenditure and dysfunctional BAT. In a series of in vitro and in vivo experiments using p62^Δ69-251^ mice, global p62-deficient mice (p62^−/−^) and Ucp1-Cre p62^flx/flx^ mice, we demonstrate that p62 is a key signaling node of the UCP1 pathway. p62 directly binds to ATF2 to orchestrate its genomic binding to and activation of the *Ucp1* enhancer and *Pgc-1α* promoter. As demonstrated in p62^Δ69-251^ mice, global p62^−/−^ mice and Ucp1-Cre p62^flx/flx^ mice, lack of p62 action leads to failure of ATF2 to activate its nuclear targets Ucp1 and Pgc1*α* and results in impaired BAT function and increased body weight. The cell autonomous effect of p62 to modulate ATF2 nuclear target activation is confirmed in BAT primary cells from p62^Δ69-251^ mice and is verified in cultured BAT cells of global p62^−/−^ mice and Ucp1-Cre p62^flx/flx^ mice. Our data establish p62 as a key regulator of adaptive thermogenesis in that it regulates the UCP1 pathway via modulation of ATF2 genomic target activation.

## Results

### Generation of p62^Δ69-251^ mice

To dissect the role of p62 in regulating systems metabolism, we generated mice in which the amino acids 69–251 of the p62 protein have been deleted (p62^Δ69-251^ mice). Mice were bred on the C57BL/6J background and were designed to yield a truncated p62 protein of ∼37 kDa that lacks the zinc finger (zz) domain, the TB1 domain and one of the two p38 interacting motifs, but to otherwise maintain regular p62 function (Supplementary Fig. 1a). Consistent with this, we see no difference in protein levels of phosphorylated protein kinase C (p-PKC) in the liver (Supplementary Fig. 1b) and of p-ERK1/2 in WAT of p62^Δ69-251^ mice (Supplementary Fig. 1c, d). Also, hepatic protein levels of microtubule-associated protein 1 light chain 3 (LC3) are, as expected, unchanged in p62^Δ69-251^ mice (Supplementary Fig. 1e), which is consistent with demonstration of preserved p62 binding to LC3 and to p38 using immunoprecipitation analysis in HEK293FT cells (Supplementary Fig. 1f). Notably, preserved p62 binding to p38 is not unexpected given that only one of the two p38 binding motifs is deleted in the p62^Δ69-251^ mice (Supplementary Fig. 1a) and no difference in p38/p-p38 is observed in BAT of p62^Δ69-251^ mice and global p62^−/−^ mice (Supplementary Fig. 1g). We further see, as expected, no difference in autophagy, as assessed by hepatic protein level of ATG7 (Supplementary Fig. 1h), or the proteasome activity in BAT of p62^Δ69-251^ mice (Supplementary Fig. 1i). Collectively, these data indicate that the p62 motifs not affected by the p62^Δ69-251^ mutation remain functional.

### p62^Δ69-251^ mice are obese with no difference in adipogenesis

**Fig. 1:**
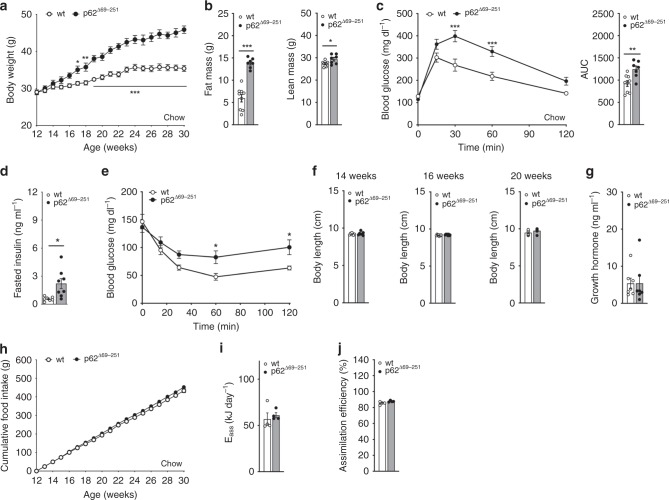
Metabolic phenotype of chow-fed male p62^Δ69-251^ mice. Body weight development (**a**) as well as body composition (**b**) and glucose tolerance (**c**) in 31-wk-old male C57Bl/6J wt or p62^Δ69-251^ mice. Fasted insulin levels (**d**) and insulin sensitivity (**e**) in 24-wk-old male mice. Body length at the age of 14, 16, or 20 wks (**f**). Growth hormone at the age of 14 wks (**g**). Cumulative food intake (**h**) as well as assimilated energy (**i**) and assimilation efficiency (**j**) at the age of 30 wks. Sample sizes are (**a**–**c**) *n* = 9/7 mice, (**d**, **e**) *n* = 7/8 mice, (**f**) *n* = 7/7 mice, *n* = 8/8 mice, *n* = 8/8 mice, (**g**) *n* = 7/7 mice. Cumulative food intake (**h**) was assessed per cage in *n* = 6/4 cages containing *n* = 9/7 mice. For panels (**i**) and (**j**), feces were collected per cage in *n* = 4/4 cages containing *n* = 8/8 mice. Data represent mean ± SEM. Longitudinal data in panels (**a**), (**c**), (**e**), and (**h**) were analyzed using two-way ANOVA with time and genotype as co-variant and using Bonferroni post hoc multiple comparison analysis for differences at individual time points. Bar graphs in panels (**b**), (**c**), (**d**), (**f**), (**g**), (**i**), and (**j**) were analyzed using two-sided two-tailed *t*-test. *p < 0.05; **p < 0.01; ***p < 0.001. Exact *p*-values are (**a**) d17 *p* = 0.0318, d18 *p* = 0.0125, d19 *p* < 0.0001, d20 *p* = 0.0002, d21–d30 *p* < 0.0001; (**b**) *p* = 0.000001996 and *p* = 0.0228; (**c**) 30 min *p* < 0.0001, 60 min *p* = 0.0004; AUC *p* = 0.00467; (**d**) *p* = 0.0132; (**e**) 60 min *p* = 0.0237, 120 min *p* = 0.0358. AUC area under curve, *E*_ass_ assimilated energy.

p62^Δ69-251^ mice develop an obese phenotype of identical dimension relative to global and adipose-specific p62^−/−^ mice when fed with either regular chow or a high-fat diet (HFD) (Fig. 1a, Supplementary Fig. 2). Since p-ERK1/2 is not changed in WAT of either 12- or 32-wk-old p62^Δ69-251^ mice (Supplementary Fig. 1c, d), ERK1/2 mediated adipogenesis can thus not explain the obese phenotype of the p62^Δ69-251^ mice. Consistent with this, we see adipocyte size and volume increased but the total number of adipocytes decreased in the iWAT, eWAT, and rpWAT of young 14-wk-old non-obese p62^Δ69-251^ mice (Supplementary Fig. 3a-d). In none of the analyzed white fat depots (Supplementary Fig. 4), nor in iWAT or BAT primary cells of p62^Δ69-251^ mice (Supplementary Fig. 5c-f, i-l), do we see major expression differences indicative of enhanced adipogenesis or adipocyte differentiation (*Pparg*, *Fasn*, *Fabp4*, *Adipoq*). This is further supported by unaltered Oil Red O staining during differentiation of BAT and iWAT primary cells (Supplementary Fig. 5a,b,g,h). These data collectively show that obesity in p62^Δ69-251^ mice does not result from changes in adipogenesis.

Obesity in chow-fed p62^Δ69-251^ mice (Fig. 1a) is primarily the result of increased body fat with only a slight (but significant) increase in lean tissue mass (Fig. 1b), and is accompanied by glucose intolerance (Fig. 1c), elevated fasting levels of insulin (Fig. 1d) and insulin resistance (Fig. 1e). The increased adiposity is not related to changes in body length (Fig. 1f) or to circulating levels of growth hormone (Fig. 1g), nor to alterations in food intake (Fig. 1h) or the amount of nutrients that are made available for metabolism (Fig. 1i, j). Since intake and absorption of nutrients is unchanged in the p62^Δ69-251^ mice, these data suggest that obesity in p62^Δ69-251^ mice results from alterations in energy expenditure.

### Obese p62^Δ69-251^ mice have an impaired BAT function

**Fig. 2:**
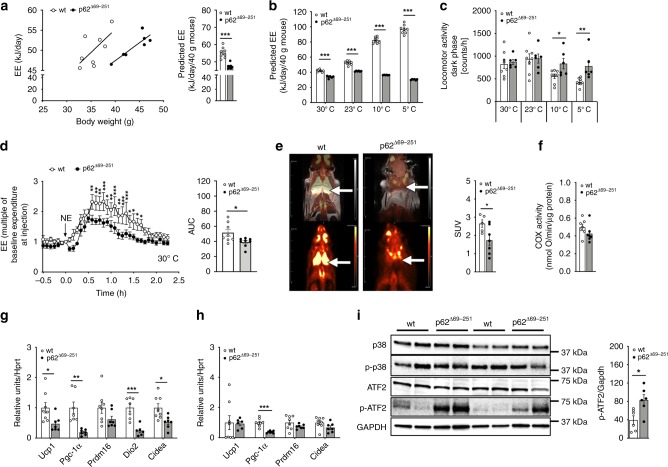
Impaired energy expenditure and BAT thermogenesis in obese p62^Δ69-251^ mice. Energy expenditure (EE) plotted against body weight and ANCOVA predicted energy expenditure at a given body weight of 40 g in obese 32-wk-old male C57Bl/6J wt or p62^Δ69-251^ mice fed a regular chow diet. When considering body weight as covariate, the ANCOVA genotype effect is *p* = 0.016 (**a**). Predicted energy expenditure (**b**) and locomotor activity during the dark phase (**c**) of 26-wk-old male mice at different environmental temperatures. Energy expenditure of male mice during a norepinephrine (NE) challenge at 30 °C (**d**). PET/MRI imaging of male 8-mths-old male mice with standardized uptake values (SUV); white arrow indicates location of BAT (**e**). Cytochrome *c* oxidase (COX) activity normalized to protein concentration (**f**), thermogenic gene expression profiling in BAT (**g**) and iWAT (**h**), and protein levels of p-p38, p38, ATF2, p-ATF2 (Thr69/71), and GAPDH (**i**) in BAT of obese 33-wk-old male mice. Samples sizes are (**a**) *n* = 8/7 mice, (**b**, **c**) *n* = 8/6 mice, (**d**) *n* = 8/8 mice, (**e**) *n* = 5/7 mice, (**f**) *n* = 7/7 mice, (**g**) *Ucp1* *n* = 8/7 mice; *Pgc-1α* *n* = 7/7 mice, *Prdm16* *n* = 8/7 mice, *Dio2* *n* = 7/6 mice, *Cidea* *n* = 8/7 mice, (**h**) *Ucp1* *n* = 8/6 mice, *Pgc-1α* *n* = 8/7 mice, *Prdm16* *n* = 8/7 mice, *Cidea* *n* = 8/7 mice, (**i**) representative western blot of *n* = 4 (out of *n* = 6) p62 wt and *n* = 4 (out of *n* = 6) p62^Δ69-251^ mice and quantification of pATF2/GAPDH in *n* = 6 mice each genotype. The full blot with *n* = 6 mice each genotype is provided in the Source data file. Data represent mean ± SEM. Data in panels (**a**) and (**b**) were analyzed using ANCOVA using body weight as covariate. Bar graphs in panels (**a**), (**b**), (**c**), (**e**), (**f**), (**g**), (**h**), and (**i**) were analyzed using two-side two-tailed *t*-test. Data in (**d**) were analyzed using two-way ANOVA using time and genotype as covariates and with Bonferroni post hoc multiple comparison test for each individual time-point. *p < 0.05; **p < 0.01; ***p < 0.001. Exact *p*-values are given in the Source data file. Hprt hypoxanthine guanine phosphoribosyltransferase, Prdm16 PR/SET domain 16, Cidea cell death-inducing DNA fragmentation factor alpha subunit-like effector A, Dio2 deiodinase 2, GAPDH Glyceraldehyde-3-phosphate dehydrogenase.

Upon ANCOVA correction for body weight^18^, obese p62^Δ69-251^ mice have strikingly decreased energy expenditure (Fig. 2a). Furthermore, p62^Δ69-251^ mice are cold intolerant and fail to appropriately increase their energy expenditure with decreasing environmental temperature (Fig. 2b). While locomotor activity is not different at either 30 or 23 °C, p62^Δ69-251^ mice increase their dark phase activity when exposed to either 10 or 5 °C (Fig. 2c). Consistent with failure of the obese p62^Δ69-251^ mice to increase their energy expenditure when exposed to cold (Fig. 2b), we see a blunted increase in energy expenditure when p62^Δ69-251^ mice are treated with norepinephrine (NE) (Fig. 2d). These data suggest that the impaired energy expenditure of p62^Δ69-251^ mice resides from inadequate activation of the BAT and that the p62^Δ69-251^ mice increase their locomotor activity upon cold exposure to compensate for BAT dysfunction. Consistent with this, BAT glucose uptake, measured by positron emission tomography/magnetic resonance imaging (PET/MRI) following administration of ^18^F-FDG, is reduced in p62^Δ69-251^ mice (Fig. 2e) despite normal activity of cytochrome *c* oxidase (COX) and thus functional mitochondrial complex IV (Fig. 2f). The impaired BAT function of p62^Δ69-251^ mice is associated with decreased expression of the key thermogenic genes *Ucp1*, *Pgc-1α*, diodinase 2 (*Dio2*), and cell death-inducing DNA fragmentation factor alpha subunit-like effector A (*Cidea*) in BAT (Fig. 2g). Expression levels *Pgc-1α* also decreased in iWAT of obese p62^Δ69-251^ mice with very low to borderline expression of *Ucp1* in both groups (Fig. 2h). Notably, despite ~50% decreased expression of *Ucp1* and ~80% decreased expression of *Pgc-1α* in the BAT (Fig. 2g), we see protein levels of the upstream *Ucp1* and *Pgc-1α* key activator p-ATF2 (Thr69/71) increased in BAT of obese p62^Δ69-251^ mice, with unchanged levels of p38 and p-p38 (Fig. 2i). Since p-ATF2 induces expression of *Ucp1* and *Pgc-1α* via direct binding to CRE elements in the promoter/enhancer regions of these genes^11^, these data suggest that p-ATF2 requires a critical p62 motif either for nuclear translocation, or for binding and activating the CRE elements of the *Ucp1* enhancer and *Pgc-1α* promoter.

### Impaired BAT function in young non-obese p62^Δ69-251^ mice

**Fig. 3:**
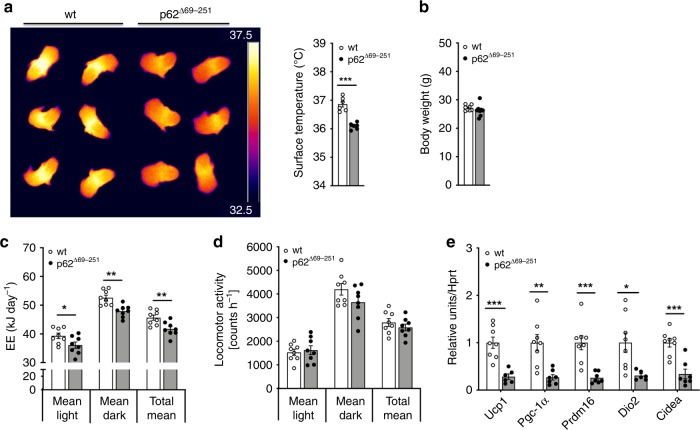
Impaired energy expenditure and BAT thermogenesis in lean p62^Δ69-251^ mice. Infrared thermographic imaging and surface temperature of 7-d-old male wt or p62^Δ69-251^ mice (**a**). Body weight (**b**), energy expenditure (**c**), and locomotor activity (**d**) of lean, non-obese 12-wk-old male mice fed with a regular chow diet. Gene expression of thermogenic markers in BAT of lean non-obese 12-wk-old male mice (**e**). Sample sizes are (**a**) *n* = 6/6 mice, (**b**–**d**) *n* = 8/8 mice, (**e**) *Ucp1* n = 8/6 mice, *Pgc-1α* *n* = 8/7 mice, *Prdm16* *n* = 8/7 mice, *Dio2* *n* = 8/6 mice, *Cidea* *n* = 8/7 mice. Data represent mean ± SEM. Bar graphs were analyzed using two-sided two-tailed *t*-test. *p < 0.05; **p < 0.01; ***p < 0.001. Exact *p*-values are (**a**) *p* = 0.000037; (**b**) *p* = 0.327; (**c**) mean light *p* = 0.0425, mean dark *p* = 0.00128, mean total *p* = 0.0068; (**d**) mean light *p* = 0.691 mean dark *p* = 0.135, mean total *p* = 0.352; (**e**) *Ucp1* *p* = 0.000383, *Pgc1a* *p* = 000217, *Prdm16* *p* = 0.00064, *Dio2* *p* = 0.0141, *Cidea* *p* = 0.00029.

If p62 is required for adequate BAT function, then lack of p62 signaling should already affect BAT thermogenesis before the onset of obesity. In line with this notion, we see BAT temperature and whole-surface temperature already decreased in 7-d-old p62^Δ69-251^ pups relative to wt controls, as assessed by infrared thermography (Fig. 3a). Consistent with this, in 12-wk-old non-obese p62^Δ69-251^ mice we see, despite yet unchanged body weight (Fig. 3b), energy expenditure already decreased (Fig. 3c). Similar to the obese p62^Δ69-251^ mice, locomotor activity is unchanged under room temperature also in the young non-obese p62^Δ69-251^ mice (Fig. 3d), but expression of key thermogenic genes *Ucp1*, *Pgc-1α*, PR/SET domain 16 (*Prdm16*), *Dio2*, and *Cidea* are decreased in BAT (Fig. 3e). These data imply that the abrogated BAT function is a direct result of p62 deficiency and is not a consequence of the obese phenotype. Consistent with this, gene signatures indicative of decreased thermogenesis (*Ucp1*, *Pgc-1α*, *Prdm16*) are confirmed also in BAT and iWAT primary cells harvested from young non-obese p62^Δ69-251^ mice (Supplementary Fig. 6a-h).

In line with the notion that young non-obese p62^Δ69-251^ mice already have an energy surplus due to decreased energy expenditure (Fig. 3c), we see histological differences in the adipose tissue even before their body weight separates from the wt controls. Accordingly, at the age of 14 wks, the p62^Δ69-251^ mice show enhanced lipid accumulation in BAT, iWAT, eWAT, and rpWAT with greater abundance of large adipocytes in all analyzed fat depots (Supplementary Fig. 3a-d) and increased weight of the iWAT and eWAT but not yet of the BAT and rpWAT (Supplementary Fig. 7a-g). Also levels of triglycerides but not cholesterol are already increased in the liver of these young non-obese p62^Δ69-251^ mice (Supplementary Fig. 7h,i).

### Impaired BAT function in Ucp1-Cre p62^flx/flx^ mice

**Fig. 4:**
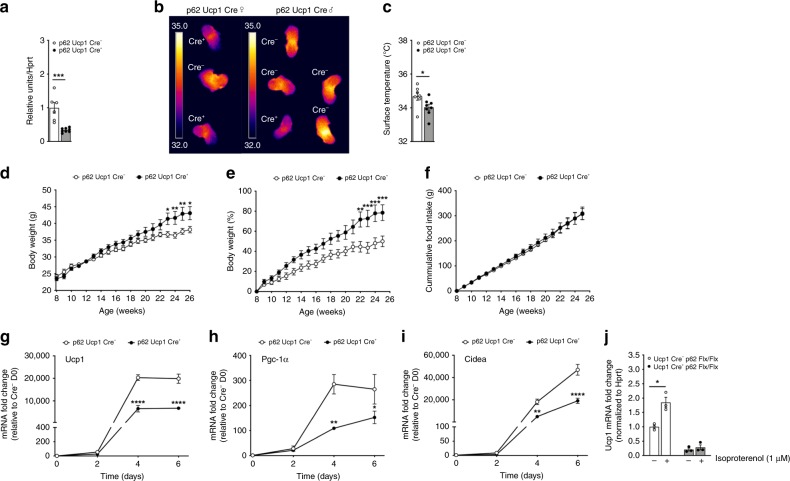
Impaired BAT function in p62^flx/flx^ Ucp1-Cre mice. Expression of p62 in BAT of p62^flx/flx^ Ucp1-Cre negative (Cre−) or p62^flx/flx^ Ucp1-Cre positive (Cre+) mice (**a**), infrared thermographic imaging in female and male mice (**b**) and surface temperature (**c**) of 7-d-old p62^flx/flx^ Ucp1-Cre− or p62^flx/flx^ Ucp1-Cre+ mice. Body weight (**d**), body weight change (**e**), and cumulative food intake (**f**) of HFD-fed p62^flx/flx^ male Ucp1-Cre− or p62^flx/flx^ Ucp1-Cre+ mice. Gene expression of *Ucp1* (**g**), *Pgc-1α* (**h**) and *Cidea* (**i**) and isoproterenol-induction of Ucp1 mRNA expression (**j**) in BAT primary cells harvested from 6- to 8-wk-old male p62^flx/flx^ Ucp1-Cre− or p62^flx/flx^ Ucp1-Cre+ mice, during 6 d of differentiation. Sample sizes are (**a**) *n* = 6/8 mice, (**b**) *n* = 3/5 mice, (**c**–**e**) *n* = 7/8 mice. **f** cumulative food intake was assessed per cage in *n* = 4/4 cages containing *n* = 7/8 mice; 2 cages (*n* = 1/1) were excluded because mice shredded food. **g**–**i** BAT primary cells were harvested from p62 wt or Ucp1-cre p62^flx/flx^ mice, plated with equal number of cells in *n* = 3 wells each genotype and individually differentiated into mature brown adipocytes. Target gene expression was then measured in *n* = 3 individually differentiated wells each genotype with two technical replicates each well. **j** BAT primary cells were harvested from p62 wt or Ucp1-cre p62^flx/flx^ mice, plated with equal number of cells in *n* = 3 wells each treatment group and individually differentiated into mature brown adipocytes. Target gene expression was then measured in *n* = 3 individually differentiated wells each treatment group with two technical replicates each well. Data in (**d**–**i**) are analyzed using two-way ANOVA using time and genotype as covariate and using Bonferroni post hoc multiple comparison to test for differences in individual time points. Data in (**a**), (**c**), and (**j**) were analyzed using two-sided two-tailed *t*-test. Data represent mean ± SEM. *p < 0.05; **p < 0.01; ***p < 0.001; ****P < 0.0001. Exact *p*-values are (**a**) *p* = 0.000424; (**c**) *p* = 0.0428; (**d**) d23 *p* = 0.0286, d24 *p* = 0.0082, d25 *p* = 0.0070, d26 *p* = 0.150; (**e**) d22 *p* = 0.0019, d23 *p* = 0.0006, d24 *p* = 0.0004, d25 *p* = 0.0009; (**g**) d4 *p* < 0.0001, d6 *p* < 0.0001; (**h**) d4 *p* = 0.0011, d6 *p* = 0.037; (**i**) d4 *p* = 0.0015, d6 *p* < 0.00; (**j**) *p* = 0.0116.

To corroborate the direct role of p62 to regulate BAT function in vivo, we generated mice with targeted deletion of p62 specifically in the BAT (Ucp1-Cre p62^flx/flx^). In BAT of these mice, expression of p62 is decreased by ~75% relative to wt controls (Fig. 4a). Despite showing 25% residual expression of p62 in BAT, these Ucp1-Cre p62^flx/flx^ mice show decreased BAT surface temperature after birth (Fig. 4b, c) and show a greater body weight gain relative to wt controls (Fig. 4d, e). Notably, while mice with global p62 deletion^15^, WAT/BAT-specific p62 deletion^16^, global p62^Δ69-251^ mutation (Fig. 2a, Supplementary Fig. 2a,d), and BAT-specific p62 deletion (Fig. 4) all become obese with impaired BAT function, mice with CNS-specific p62 deletion do not become obese^16^. These data collectively demonstrate that the metabolic dysfunction residing in p62 deficiency originates from p62 signaling in the BAT and is independent of p62 signaling in the CNS. Consistent with the p62^Δ69-251^ mice, also the increased body weight of the Ucp1-Cre p62^flx/flx^ mice is not accompanied by changes in food intake (Fig. 4f), but expression of key thermogenic genes (*Ucp1*, *Pgc-1α, Cidea*) are decreased in in vitro cultured brown adipocytes of the Ucp1-Cre p62^flx/flx^ mice (Fig. 4g–i). In line with these data, isoproterenol fails to induce *Ucp1* mRNA levels in BAT primary cells of the Ucp1-Cre p62^flx/flx^ mice (Fig. 4j).

### p62 regulation of BAT function is cell autonomous

**Fig. 5:**
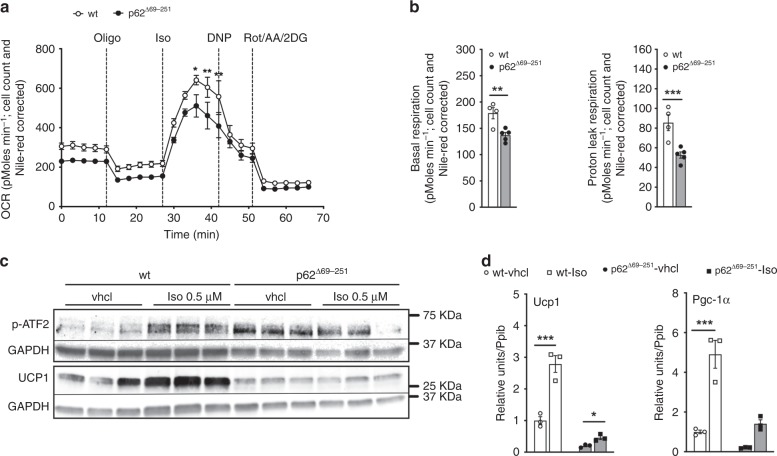
Cell autonomous regulation of energy expenditure by p62. Oxygen consumption rate (**a**), as well as basal and proton leak respiration (**b**) in BAT primary cells harvested from 6- to 8-wk-old male wt or p62^Δ69-251^ mice. Western blot analysis of p-ATF2 (Thr69/71) and UCP1 (**c**) and gene expression of *Ucp1* and *Pgc-1α* (**d**) in 6 d differentiated BAT primary cells harvested from 6- to 8-wk-old male wt or p62^Δ69-251^ mice and which have been stimulated with saline (vhcl) or isoproterenol (0.5 μM) for 6 h. For measurement of oxygen consumption (**a**), basal respiration (**b**) and proton leak respiration (**c**), BAT primary cells were harvested from p62 wt or p62^Δ69-251^ mice, plated with equal number of cells in *n* = 4/5 wells each genotype and individually differentiated into mature brown adipocytes. Oxygen consumption was then measured in *n* = 4/5 individually differentiated wells each genotype with *n* = 3 technical replicates each well. For data in panel (**c**), BAT primary cells were harvested from p62 wt or p62^Δ69-251^ mice, plated with equal number of cells in *n* = 3 wells each treatment group and individually differentiated into mature brown adipocytes. Protein level of p-ATF2, GAPDH and UCP1 was then measured in *n* = 3/3/3/3 individually differentiated wells. For data in panel (**d**), BAT primary cells were harvested from p62 wt or p62^Δ69-251^ mice, plated with equal number of cells in *n* = 3 wells each treatment group and individually differentiated into mature brown adipocytes. Target gene expression was then measured in *n* = 3/3/3/3 individually differentiated wells with *n* = 2 technical replicates each well. Panels (**a**) and (**d**) are representative examples of two independently performed studies, each yielding similar results. Data in panel (**a**) have been analyzed using two-way ANOVA with time and genotype as co-variant and using Bonferroni post hoc multiple comparison to test for differences in individual time points. Bar graphs have been analyzed using two-sided two-tailed *t*-test. Data represent mean ± SEM. *p < 0.05; **p < 0.01; ***p < 0.001. Exact *p*-values are given in the Source data file.

We also assessed energy expenditure using Seahorse analysis and see basal respiration and proton leak respiration decreased in BAT primary cells of p62^Δ69-251^ mice (Fig. 5a, b). In agreement with our observation that p-ATF2 is increased and expression of *Ucp1* decreased in the p62^Δ69-251^ mice (Fig. 2g, i), we see increased levels of p-ATF2, yet lower protein and transcript levels of *Ucp1* also in BAT primary cells of p62^Δ69-251^ mice; even when cells are stimulated with isoproterenol (Fig. 5c, d). These in vitro data again suggest that p-ATF2 requires functional p62 to access or activate the CRE elements in the promoter/enhancer region of *Ucp1* and *Pgc-1α*, and they consequently further demonstrate that the effects of p62 on BAT function are cell autonomous and are not driven by CNS effects.

### p62 regulates ATF2 nuclear target activation

**Fig. 6:**
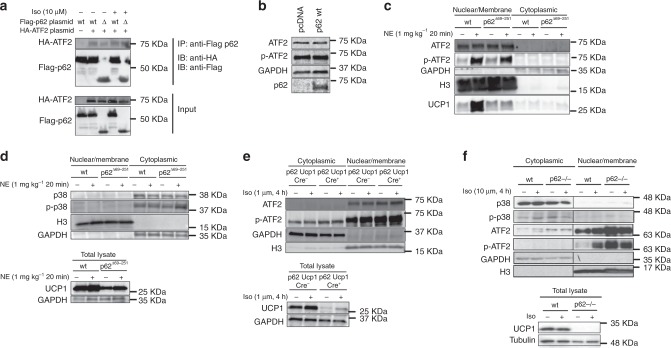
p62 regulation of ATF2 nuclear target activation. Co-immunoprecipitation (IP) in HEK293T cells transfected with Flag-p62 wt, Flag-p62^Δ69-251^, or HA-ATF2, respectively, and stimulated with isoproterenol as indicated. Flag-IP was performed to pulldown p62. Western blot analysis of pulldown (upper image) and input fraction (lower image) (**a**). Western blot analysis of ATF2 and p-ATF2 upon p62 overexpression in primary brown adipocytes isolated from 8-wk-old male p62^Δ69-251^ mice (**b**). Nuclear/membrane and cytosolic protein level of ATF2, p-ATF2 (Thr69/71), GAPDH, Histone 3 (H3), and UCP1 in BAT of 12-wk-old male wt and p62^Δ69-251^ mice upon injection of norepinephrine (1 mg kg^−1^) for 20 min (**c**). Nuclear/membrane and cytosolic protein level of p38, p-p38, GAPDH, Histone 3 (H3) as well as total lysates showing UCP1 in BAT of male wt and p62^Δ69-251^ mice upon injection of norepinephrine (1 mg kg^−1^) for 20 min (**d**). Nuclear/membrane and cytosolic protein level of ATF2, p-ATF2 (Thr69/71), GAPDH, Histone 3 (H3) as well as total lysates showing UCP1 upon 4 h stimulation with 1 µM isoproterenol in immortalized brown adipocytes from 6- to 8-wk-old male p62^flx/flx^ Ucp1-Cre− or p62^flx/flx^ Ucp1-Cre+ mice (**e**). Nuclear/membrane and cytosolic protein level of p38, p-p38, ATF2, p-ATF2 (Thr69/71), GAPDH, Histone 3 (H3) as well as total lysates showing UCP1 upon 4 h stimulation with 10 µM isoproterenol in immortalized brown adipocytes from wt or p62^−/−^ mice (**f**). Panel (**a**) represents a representative example of three independently performed studies, each yielding similar results. Panels (**b**), (**c**), (**d**), and (**f**) are representative examples of two independently performed studies, each yielding similar results.

**Fig. 7:**
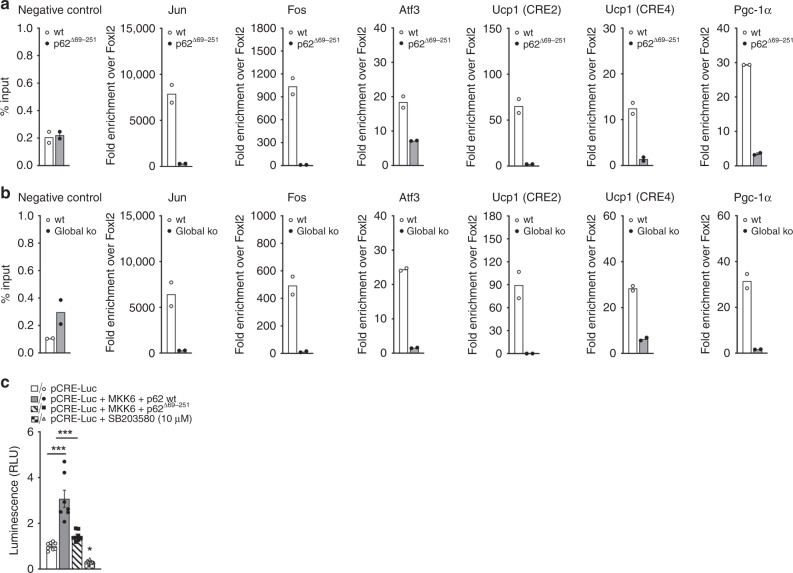
Impaired genomic binding of ATF2 in p62^Δ69-251^ and global p62^−/−^ mice. Chromatin immunoprecipitation (ChIP) in BAT of male wt and p62^Δ69-251^ mice (**a**) and p62^−/−^ mice (**b**) using antibodies specific for ATF2. ChIP-qPCR analysis of *Foxl2* (negative control), *Jun*, *Fos*, *Atf3*, *Ucp1 (CRE2)*, *Ucp1 (CRE4)*, and *Pgc-1α* (*n* = 5 mice pooled each genotype, *n* = 2 technical replicates) (**a**, **b**). Luciferase-based CRE reporter assay of HEK293T cells transfected with the pCRE-Luc reporter, a dominant positive MKK6 (K82A) and p62 wt or p62^Δ69-251^ (*n* = 7/8 technical replicates each group) (**c**). Panel (**c**) is a representative example of three independently performed studies, each yielding similar results. Data in (**a**) and (**b**) have been analyzed using a two-sided two-tailed *t*-test, data in (**c**) have been analyzed using one-way ANOVA was treatment as co-variant and using Bonferroni multiple comparison between the treatment groups. Data represent mean ± SEM. *p < 0.05; **p < 0.01; ***p < 0.001. Exact *p*-values are (**c**) pCRE-Luc vs. pCRE-Luc + MKK6 + p62 wt *p* < 0.0001, pCRE-Luc vs. pCRE-Luc + MKK6 + p62^Δ69-251^ *p* > 0.05, pCRE-Luc vs. pCRE-Luc + MKK6 + SB203580 *p* > 0.05, pCRE-Luc + MKK6 + p62 wt vs. pCRE-Luc + MKK6 + p62^Δ69-251^ *p* < 0.0001, pCRE-Luc + MKK6 + p62 wt vs. pCRE-Luc + MKK6 + SB203580 *p* > 0.0001, pCRE-Luc + MKK6 + p62^Δ69-251^ vs. pCRE-Luc + MKK6 + SB203580 *p* = 0.0005. Foxl2 forkhead box L2, Fos FBJ osteosarcoma oncogene, Atf3 activating transcription factor 3.

We next tested whether p62 is a direct ATF2 interaction partner and whether p62 is required either for ATF2 nuclear translocation or for binding to and activation of its nuclear targets. Immunoprecipitation analysis in HEK293T cells showed direct binding of p62 to ATF2 (Fig. 6a) without ability of p62 to directly activate (phosphorylate) ATF2 (Fig. 6b). Notably, p62 binding to ATF2 is observed under both baseline conditions and after stimulation with isoproterenol and is preserved even in the p62^Δ69-251^ mutants (Fig. 6a). We next tested whether p62 is required for nuclear translocation of ATF2 and quantified the cytosolic and nuclear content of ATF2. In line with the observation that p62 binding to ATF2 is preserved in p62^Δ69-251^ mutant cells (Fig. 6a), nuclear presence of p-ATF2 is preserved in BAT of the p62^Δ69-251^ mice under both baseline conditions and after β-adrenergic receptor stimulation (Fig. 6c). Yet, despite normal activation (phosphorylation) and nuclear presence of ATF2 in response to β-adrenergic receptor stimulation, p-ATF2 fails to increase protein levels of UCP1 in the p62^Δ69-251^ mice (Fig. 6c, d). Preservation of p-ATF2 nuclear entry with failure to induce UCP1 levels are confirmed also in BAT primary cells of Ucp1-Cre p62^flx/flx^ mice (Fig. 6e) and in BAT cells of global p62^−/−^ mice (Fig. 6f). Consistent with the observation that protein level of p38 and p-p38 are unchanged in whole BAT lysates of the p62^Δ69-251^ mice and the global p62^−/−^ mice (Fig. 2i and Supplementary Fig. 1g), we see levels of p38 and p-p38 also unchanged in the nuclear/cytosolic fractions of BAT in young non-obese p62^Δ69-251^ mice (Fig. 6d) and this is confirmed also in cultured BAT cells of the global p62^−/−^ mice (Fig. 6f). Collectively, these data indicate that the observed changes in p-ATF2 are not related to changes in p38 and further suggest that functional p62 is required for ATF2 to bind and activate its nuclear targets. We next performed chromatin immunoprecipitation (ChIP) assays in BAT of the p62^Δ69-251^ mice. There was abundant endogenous genomic binding of ATF2 at canonical target sites in BAT from wt mice (Fig. 7a). These ATF2 target sites include promoters of *Jun*, *Fos*, and *Atf3*^19^, as well as previously described BAT-specific ATF2 target sites within the *Ucp1* enhancer (CRE2) and promoter (CRE4), and the *Pgc-1α* promoter^11^. Importantly, ATF2 binding to target loci was severely impaired in BAT from p62^Δ69-251^ mice (Fig. 7a) and this was confirmed also in BAT from global p62^−/−^ mice (Fig. 7b). These data demonstrate that failure of ATF2 to induce *Ucp1* and *Pgc-1α* expression in the p62 mutant mice is associated with impaired genomic binding of ATF2 to the promoter/enhancer regions of target genes, and implies that functional p62 is required for ATF2-mediated transcriptional activation. Consistent with a previously described transcriptional co-regulator role for p62^20–22^, we observed also reduced chromatin binding of p62 to the promoter/enhancer regions of *Ucp1* and *Pgc-1α* in BAT of the p62^−/−^ mice, and this was also confirmed in the BAT-specific p62 ko mice (Supplementary Fig. 8a,b). To further validate this finding, we assessed ATF2 target activation in vitro using a luciferase-based CRE reporter assay. We co-transfected HEK293T cells with an ATF2 sensor (pCRE-luc), along with the dominant positive p38/ATF2 activator MKK6(K82A) and the respective wt p62 or mutant p62^Δ69-251^. Consistent with our finding that lack of p62 leads to impaired binding of ATF2 to target genes (Fig. 7a, b), there was reduced transcriptional activation in cells transfected with the p62^Δ69-251^ plasmids relative to the p62 wt controls (Fig. 7c). Collectively, these data demonstrate that a motif encoded by the p62 amino acids 69–251 is required for ATF2 to bind and activate its nuclear targets, and suggests that its absence in the p62^Δ69-251^ mice leads to impaired energy expenditure and BAT dysfunction due to failure of ATF2 to activate key thermogenic targets in the nucleus, including the promoter/enhancer regions of *Ucp1* and *Pgc-1α*. Notably, while BAT function is severely impaired in all three analyzed p62 mutant mouse models, no ATF2-mediated transcriptional changes are observed on stress and DNA damage, ER stress, inflammation, cell cycle, cell death, and autophagy in either liver, muscle, kidney, and spleen of p62^Δ69-251^ mice (Supplementary Figs. 9,10).

## Discussion

We here demonstrate that the scaffold protein p62 is a key regulator of the UCP1 pathway. Our data demonstrate that p62 regulates the UCP1 pathway in a cell autonomous manner by directly binding to ATF2 and by regulating its binding to the *Ucp1* enhancer and *Pgc-1α* promoter. As demonstrated in p62^Δ69-251^ mice, global p62^−/−^ mice and Ucp-Cre p62^flx/flx^ mice, lack of p62 action results in severely impaired BAT function. We attribute this to failure of ATF2 to bind and activate the promoters/enhancers of its downstream thermogenic targets *Ucp1* and *Pgc-1α*. These data are consistent with the observation that lack of p62 in adipocytes, but not in the liver, muscle, myeloid lineage cells or the CNS, results in impaired BAT function and an obese phenotype^16^. A recent report suggested that the obese phenotype of p62 deficiency originates from the lack of p62 action in the CNS, leading to hyperphagia and leptin resistance^23^. However, while changes in energy expenditure are consistently observed in mice lacking p62 either globally^15^ or in an adipose-specific manner^16^, no major difference in food intake has been observed in any of these mice. Consistent with this, we also report here that p62^Δ69-251^ mice have impaired energy expenditure but no difference in food intake. Moreover, also CNS-specific p62^−/−^ mice have no obese phenotype and normal food intake^16^. Also, as occurs in the adipocyte-specific p62^−/−^ mice, the impaired energy expenditure of p62^Δ69-251^ mice cannot be explained by changes in locomotor activity. Since BAT thermogenesis is the only observed dysfunctional component of the energy balance equation, we consequently conclude that impaired energy expenditure of BAT is the causal factor leading to increased body weight in the p62-deficient mice.

We also assessed whether p62^Δ69-251^ mice have ATF2 mediated disturbances in other metabolic processes, like stress and DNA damage, ER stress, inflammation, cell cycle, cell death, and autophagy. None of these pathways seem overtly changed in either liver, muscle, kidney, and spleen of p62^Δ69-251^ mice. Notably, the impaired energy expenditure of p62^Δ69-251^ mice becomes most apparent when the mice are exposed to cold; when the BAT is most active. In light of this notion, it seems possible that other ATF2 signaling events only become apparent in the p62 mutant mice after specific stimuli. In summary, we demonstrate that the scaffold protein p62 is an essential key regulator of adaptive thermogenesis and BAT function in that it regulates *Ucp1* and *Pgc-1α* via modulation of ATF2 genomic binding. On an important note, the frequently observed increase in p-ATF2 in BAT of the p62 mutant mice does not seem to be an integral part of the pathological mechanism leading to the impaired BAT function. Accordingly, while we consistently see across all three analyzed p62 mutant mouse lines that p-ATF2 fails to activate its nuclear targets *Ucp1* and *Pgc-1α* (Fig. 6c, e, f), we see that levels of p-ATF2 are often (Figs. 2i, 5c, and 6e, f) but not always (Fig. 6c) increased in BAT. In summary, our data establish p62 as an important regulator of the UCP1 pathway. Additional studies are warranted to further characterize nuclear/cytosolic localization of p62, and to determine whether p62 regulates BAT function also via UCP1-independent mechanisms.

## Methods

### Animal studies

Mice were kept at a constant environment with ambient temperature set to 22 ± 2 °C with constant humidity (45–65%) and a 12 h/12 h light/dark cycle. Mice had free access to water and were fed ad libitum with either a standard chow (Type 1314, Altromin GmbH, Lage, Germany) or HFD (58% kcal fat; Research Diets, New Brunswick, NJ, USA; # D12331). Animal experiments were performed in accordance with European guidelines under permission of the local animal ethics committee of the state of Bavaria or the Regional Council Tübingen. Whole-body composition (fat and lean tissue mass) was measured using nuclear magnetic resonance technology (EchoMRI, Houston, TX, USA). For glucose and insulin tolerance test, mice were fasted for 6 h and then challenged with 1.75 g glucose per kg body weight or 0.75 U insulin (Humalog, Eli Lilly, Bad Homburg, Germany) per kg body weight. Generation of full-length p62 knock-out mouse lines p62^−/−^ and aP2-cre^+^;p62^loxp/loxp^ is indicated elsewhere^15,16^. p62^Δ69-251^ mice were generated by Prof. Andre Gessner (University of Regensburg) using a targeting vector that replaces exons two to four of p62 with a neomycin-resistance cassette. Ucp1-Cre p62^flx/flx^ mice were generated by pairing p62^flx/flx^ mice with mice expressing Cre recombinase under control of the Ucp1 promoter.

### Indirect calorimetry

Energy expenditure and home-cage activity were assessed using a climate-controlled combined indirect calorimetric system (TSE System, Bad Homburg, Germany). After acclimatization for 24 h, levels of O_2_ and CO_2_ were measured every 10 min for 4–5 days. Data for energy expenditure were analyzed using ANCOVA with body weight as covariate as previously suggested^18^. For ANCOVA analysis, energy expenditure raw data were blotted against the individual body weight. Every individual data point was then slighted up or down the group’s own regression line until all data line up at a predefined body weight of 40 g. The result of this ANCOVA correction by body weight was then plotted as a bar graph as previously suggested^18,24^.

### Cold challenge in wt and p62^Δ69-251^ mice

Body weight and body composition was measured at the beginning and end of the study. Mice were acclimatized to thermoneutrality (30 °C) for 1 day, followed by subsequent measurement of energy expenditure starting at 30 °C for another day. On Day 3, a non-shivering thermogenesis (NST) test was performed at 30 °C. For the NST test, mice were i.p. injected with a single bolus of Norepinephrine (Arterenol, Sanofi Aventis, Frankfurt, Germany; 1 mg kg^−1^) or saline control. Afterward, the temperature of the climate chamber was gradually decreased from 30 to 23 °C (2 days), to 10 °C (1 day), to 5 °C (1 day). Tissues were collected at the end of the study.

### In vivo PET and MRI studies

Animals were injected i.p. with 11.7 ± 0.3 MBq ^18^F-FDG dissolved in saline. Animals were kept awake and allowed to move freely in their home cages during ^18^F-FDG uptake for 30 min. Directly after that, the mice were euthanized with CO_2_. Static PET acquisitions (10 min) were performed using a dedicated micro-PET scanner (DPET, Siemens Preclinical Solutions, Knoxville, USA). The spatial resolution was 1.4 mm (center of field of view), the axial field of view was 12.7 cm. All PET scans were acquired with an energy window of 350–650 keV and a coincidence timing window of 3.432 ns. All major corrections were applied to the PET scans (e.g., normalization, decay & deadtime correction), but no attenuation correction was used as only mice on thin plastic beds were scanned. PET-images were reconstructed using the three-dimensional ordered-subset-expectation-maximization (OSEM3D) algorithm with a 128 × 128 matrix and a final pixel size of 0.79 × 0.79 ×0.80 mm³.

Animals were transferred on-bed to a 7-Tesla small animal MRI scanner (Clinscan or BioSpec with ParaVision 6.01, Bruker Biospin, Ettlingen, Germany) to ensure identical mouse positioning on-bed for PET and MRI acquisitions. T_2_-weighted anatomical MR-images were acquired using the following 3D-spoiled turbo spin echo sequence: matrix size: 256 × 256, FOV: 35 × 57 mm^2^, repetition time = 800 ms, echo time = 30.8 ms, slice thickness = 0.25 mm, flip angle 90°, echo train length 16.

### PET and MRI data analysis

Inveon Research Workplace (IRW v3.0, Siemens Preclinical Solutions, Knoxville, USA) was used for data analysis. PET and MRI data were co-registered for exact, MRI-guided delineation of volumes of interest (VOI), drawn manually for BAT based on the MR image. PET data were expressed as standardized uptake value (SUV), calculated as measured activity in BAT divided by injected activity and body weight.

### Infrared thermography

For surface body temperature measurement, 7-d-old pups were temporarily placed in a 6-well dish for temperature recording via a thermal imaging camera Optris, OPTPI400 (Ekomess, Heilbronn, Germany).

### Bomb calorimetry

For assessment of assimilated energy, food intake and feces production were assessed over a 5-d period. Feces and food were dessicated in a drying oven (50 °C) for bomb calorimetric combustion. The assimilated energy was then calculated by subtracting the energy lost with feces (*E*_out_) from the energy intake (*E*_in_). Chow control diet was combusted to calculate energy content of the absorbed food. Bomb calorimetric analysis was performed using a 6300 Oxygen Bomb Calorimeter (Parr Instrument Technology, IL, USA).

### Nuclear extraction

For the protein fractionation, p62^Δ69-251^ mice aged 17–19 wks were injected subcutaneously with a single bolus of norepinephrine (Arterenol, Sanofi Aventis, Frankfurt, Germany; 1 mg kg^−1^) or saline control and BAT was collected after 20 min in fractionation buffer (10 mM HEPES pH 7.9, 10 mM KCl, 0.1 mM EDTA, 0.1 mM EGTA, 0.4% NP-40, 1 mM DTT, 0.1 mM PMSF) with proteinase and phosphatase inhibitors (Thermo Fisher). After homogenization at 16 Hz for 2 min, samples were centrifuged at 750 × *g* for 5 min at 4 °C. The supernatant was collected and centrifuged at 10,000 rpm, 10 min (cytoplasmic fraction). This process was repeated four times to remove nuclear contamination. The pellet was washed with fractionation buffer and centrifuged at 750 × *g*, 5 min to remove cytoplasmic contamination at least three times. The nuclear/membrane fraction was dissolved in RIPA buffer.

### Cytochrome *c* oxidase activity measurement

Cox activity of interscapular BAT was assessed polarographically using a Clark type electrode (RANK Brothers, Cambridge, USA) and a Powerlab for data processing (ADInstruments, Colorado Springs, CO, USA). Tissue was weighed and homogenized mechanically in tissue buffer (100 mM potassium phosphate, 2 mM EDTA, 10 mM glutathione, pH 7.2, at 37 °C) using QIAGEN TissueLyser (Qiagen, Hilden, Germany). Homogenates were treated with detergent (0.1% *n*-dodecyl-β-D-maltoside) and subjected to a temperature-controlled reaction chamber containing 130 μM cytochrome *c* from horse heart (Sigma-Aldrich, Munich, Germany) and 18 mM ascorbate in measuring buffer (100 mM potassium phosphate, 5 mM EDTA, pH 7.2, at 37 °C).

### Hematoxylin and eosin (H&E) staining

Excised iWAT, rpWAT, eWat, and BAT samples were fixed in 4% (w/v) neutrally buffered formalin, embedded in paraffin, cut into 3-µm slices and a hematoxylin and eosin (H&E) staining was performed. The stained tissue sections were scanned with an AxioScan.Z1 digital slide scanner (Zeiss, Oberkochen, Germany) equipped with a ×20 magnification objective. Quantification of adipocytes was performed using ImageJ software.

### Isolation of primary adipocytes

Inguinal white adipose tissue was obtained from 6- to 8-wk-old male C57Bl/6J (Janvier Labs, Le Genest-Saint-Isle, France) mice. Fat pads were minced and digested for 40 min at 37 °C (1 mg ml^−1^ Collagenase IV (Sigma-Aldrich, Munich, Germany); 3 U/ml Dispase II (Sigma-Aldrich, Munich, Germany); 0,01 mM CaCl_2_ in PBS). Cell suspension was filtered through a 100 µm cell strainer, centrifuged (500 × *g* for 10 min) and resuspended in growth medium DMEM/F12 (1:1) plus Glutamax (Thermo Fisher Scientific, Erlangen, Germany), 1% Pen/Strep and 10% FBS after each step. Homogenate was filtered through a 70 µm cell strainer. Primary white adipocytes were plated onto collagen-coated dishes (Fisher Scientific, Schwerte Germany) and grown to confluence (37 °C, 10% CO_2_). At confluence, cells were exposed to an adipogenic cocktail containing dexamethasone (1 µM), isobutylmethylxanthine (0.5 mM), rosiglitazone (1 µM) and insulin (5 µg ml^−1^) in growth medium. After 48-h induction, cells were maintained in culture medium containing insulin (5 µg ml^−1^) and rosiglitazone (1 µM). On Day 4 of differentiation, cells were cultured in growth medium containing insulin (5 µg ml^−1^). To stimulate thermogenesis, cells were treated with 0.5 µM Isoproterenol (Sigma-Aldrich, Munich, Germany), dissolved in serum-free growth medium, for 6 h on Day 6 of adipocyte differentiation.

Primary brown adipocytes were isolated via the same procedure with the exception that 1 mg/ml Collagenase II (Sigma-Aldrich, Munich, Germany) was used for digestion. For differentiation of primary brown adipocytes, the induction cocktail contained dexamethasone (5 µM), isobutylmethylxanthine (0.5 mM), rosiglitazone (1 µM), indomethacin (125 µM), T3 (1 nM), and insulin (0.5 µg ml^−1^) in growth medium. Two days post-induction of differentiation, cells were maintained in culture medium containing rosiglitazone (1 µM), T3 (1 nM), and insulin (0.5 µg ml^−1^). On Day 4 of differentiation, cells were cultured in growth medium containing T3 (1 nM) and insulin (0.5 µg ml^−1^).

### Oil Red O staining

Cells were fixed with 4% paraformaldehyde (PFA) during different days of differentiation. Adipocytes were stained with Oil Red O (Sigma-Aldrich, Munich, Germany) for 10 min and immediately washed with H_2_O. For lipid quantification, Oil Red O was retrieved from the cells by 100% isopropanol and absorbance was measured at 500 nm. Dapi signal was measured to correct for cell number.

### Preparation of RNA and gene expression analysis

Total RNA was prepared using RNeasy Kit (Qiagen, Hilden, Germany) according to manufacturer’s instructions. cDNA synthesis was performed with QuantiTect Reverse Transcription Kit (Qiagen, Hilden, Germany) according to manufacturer’s instructions. Gene expression of cell samples (*N* = 3 per group) was profiled with quantitative PCR-based (qPCR) techniques using SYBR green or TaqMan Single Probes (Thermo Fisher Scientific, Erlangen, Germany). The relative expression of the selected genes was measured using the 7900HT Fast Real-Time PCR System (Thermo Fisher Scientific, Erlangen, Germany). The relative expression levels of each gene were normalized to the housekeeping gene peptidylprolyl isomerase B (*Ppib*) or Hypoxanthine Phosphoribosyltransferase (*Hprt*). TaqMan Low Density Array (Thermo Fisher Scientific, Erlangen, Germany) was performed according to instructions. The following primers were used: *Ucp1* forward 5ʹ-GGCCTCTACGACTCAGTCCA-3ʹ, *Ucp1* reverse 5ʹ-TAAGCCGGCTGAGATCTTGT-3ʹ, *Pgc-1α* forward 5ʹ-AGCCGTGACCACTGACAACGAG-3ʹ, *Pgc-1α* reverse 5ʹ-GCTGCATGGTTCTGAGTGCTAAG-3ʹ, *Prdm16* forward 5ʹ-CCGCTGTGATGAGTGTGATG-3ʹ, *Prdm16* reverse 5ʹ-GGACGATCATGTGTTGCTCC-3ʹ, *Bmp8b* forward 5ʹ-TCCACCAACCACGCCACTAT-3ʹ, *Bmp8b* reverse 5ʹ-CAGTAGGCACACAGCACACCT-3ʹ, *p62/Sqstm1* wild type forward 5ʹ-CCAGTGATGAGGAGCTGACA-3ʹ, *p62/Sqstm1* wild type reverse 5ʹ-CCGTTGCAACCATCACAGAT-3ʹ, *p62/Sqstm1* mutant delta 69–251 forward 5ʹ-CACTACCGCGGCATTGAG-3ʹ, *p62/Sqstm1* mutant delta 69–251 reverse 5ʹ-AGGTTTGCTGACTTCCGAAG-3ʹ. *Cidea* forward 5ʹ-AATGGACACCGGGTAGTAAGT-3ʹ, *Cidea* reverse 5ʹ-CAGCCTGTATAGGTCGAAGGT-3ʹ, Dio2 forward 5ʹ-TGCCACCTTCTTGACTTTGC-3ʹ, *Dio2* reverse 5ʹ-GGTTCCGGTGCTTCTTAACC-3ʹ, *Adiponectin* forward 5ʹ-GGTCCTAAGGGTGAGACAGG-3ʹ, *Adiponectin* reverse 5ʹ-AGTCCCGGAATGTTGCAGTA-3ʹ, *Fasn* forward 5ʹ-AGAGATCCCGAGACGCTTCT-3ʹ, *Fasn* reverse 5ʹ-GCTTGGTCCTTTGAAGTCGAAGA-3ʹ, *Fabp4* forward 5ʹ-CAGCGTAAATGGGGATTTGG-3ʹ, *Fabp4* reverse 5ʹ-CCGCCATCTAGGGTTATGAT-3ʹ, *Pparg* forward 5ʹ-TCGCTGATGCACTGCCTATG-3ʹ, *Pparg* reverse 5ʹ-GAGAGGTCCACAGAGCTGATT-3ʹ. The following taqman probes were used: *p62* Mm01070495_m1, *Ucp1* Mm01244861_m1, *Pgc-1α* Mm01208835_m1, *Dio2* Mm00515664_m1, *Cidea* Mm00432554_m1, *Bmp8b* Mm00432115_g1. *Hpr*t Mm01545399_m1, *Jun* Mm00495062_s, *Fos* Mm00487425_m1, *JunB* Mm04243546_s1, *Atf3* Mm00476033_m1, *Atm* Mm01177457_m1, *Xpa* Mm00457111_m1, *Rad23b* Mm00772280_m1, *Msh6* Mm00487761_m1, *Nos2* Mm00440502_m1, *Hspa5* Mm00517691_m1, *Ccna1* Mm01292554_m1, *Ccnd1* Mm00432359_m1, *Tnf* Mm00443258_m1, *Il8* Mm00441263_m1, *Il1b* Mm00434228_m1, *Il6* Mm00446190_m1, *Th* Mm00447557_m1, *Col1a1* Mm00801666_g1, *Mmp2* Mm00439498_m1, *Sele* Mm00441278_m1, *Selp* Mm01295931_m1, *Vcam1* Mm01320970_m1, *Ppargc1* Mm01208835_m1, *Ucp1* Mm01244861_m1, *Zfp516* Mm00813584_m1, *Mapk14* Mm01301009_m1, *Creb1* Mm00501607_m1, *Brca1* Mm00515386_m1, *Bcl2L1* Mm00437783_m1, *Ddit3* Mm01135937_g1, *Adrb2* Mm02524224_s1, *Map2k6* Mm00803694_m1, *Ep300* Mm00625535_m1, *Bcl6* Mm00477633_m1, *Nfil3* Mm00600292_s1, *Pdgfra* Mm00440701_m1, *Atf6* Mm01295319_m1, *Atf4* Mm00515325_g1, *Ulk1* Mm00437238_m1, *Atg7* Mm00512209_m1, *Atf13* Mm00521135_m1, *Map1lc3a* Mm00458724_m1.

### Bioenergetic analysis

Primary BAT cells were isolated and differentiated for 5 d on a collagen-coated XF24 well plate. On Day 5, cells were washed with DMEM XF Assay medium (Seahorse Bioscience, Santa Clara, CA, USA), supplemented with 25 mM glucose, no pyruvate and no fatty-acid free BSA. All port compounds were dissolved in pure DMEM XF Assay medium without supplements and 10-fold higher concentrated compounds were loaded into the ports of a XF Assay Cartridge. Oxygen consumption rate (OCR) was measured using an extracellular flux analyzer (XF24, Seahorse Bioscience, Santa Clara, CA, USA). Basal OCR was recorded for 15 min followed by measurement of OCR after injection of oligomycin (5 µg ml^−1^, 15 min), isoproterenol (1 µM, 15 min), 2,4-Dinitrophenol (DNP) (100 µM, 9 min), rotenone (5 µM)/antimycin (2 µM)/2-Deoxyglucose (100 mM) (15 min). For normalization, the cell plate was subsequently co-stained with Dapi and Nile red and the fluorescence signal was detected to correct for cell number and differentiation. Proton leak respiration was calculated by subtraction of Rot/AA/2DG-OCR from Oligomycin-OCR.

### Co-immunoprecipation

HEK293FT cells (ATCC, Manassas, VA, USA; #CRL-3216) or HEK293T cells (ATCC, Manassas, VA, USA; #CRL-1573) were cultured in 6-well plate and transfected with 1.5 μg Flag-p62 and 1.5 μg HA-ATF2 plasmid per well by polyethylenimine (linear, MW-25000, Polysciences, Hirschberg, Germany) for 24 h. Isoproterenol (10 μM) was added half an hour before the cells were collected. The protein lysate of 1500 μg was incubated with anti-Flag-agarose (Sigma-Aldrich, Munich, Germany) overnight for immunoprecipitation, together with 50 μg lysate as input to be loaded for western blotting detection of HA (anti-HA, Cell Signaling Technology) or Flag tag (anti-Flag). Antibodies were obtained from the following sources: Anti-HA (C29F4) (Cell Signaling, Danvers, MA, USA; #3724S; dilution: 1:1000), anti-FLAG-M2 (Sigma-Aldrich, St. Louis, MO, USA; #F1804; dilution: 1:2000), anti-beta-actin (Cell Signaling, Danvers, MA, USA #4967S; dilution: 1:2000), anti-rabbit (Sigma-Aldrich, St. Louis, MO, USA; #A3687; dilution: 1:20,000), anti-mouse (Sigma-Aldrich, St. Louis, MO, USA; #A3562; dilution: 1:20,000).

### Western blot analysis

For protein quantification, mice were euthanized by CO_2_, tissues were rapidly frozen on dry ice and stored at −80 °C until further analysis. Fat tissues were lysed in ice-cold RIPA buffer (Sigma-Aldrich, Munich, Germany) containing protease/phosphatase inhibitor (Thermo Scientific, Rockford, IL, USA), 1 mM PMSF (Carl Roth, Karlsruhe, Germany), and 10 nM Calyculin A (New England Biolabs, Frankfurt, Germany) using a polytron. Proteins were separated on Criterion gel (Bio-Rad, Munich, Germany) and transferred onto nitrocellulose membranes. Membranes were incubated with primary antibody (New England Biolabs, Frankfurt, Germany) overnight at 4 °C, and HRP-coupled secondary antibody (Santa Cruz Biotechnology Inc., Delaware, CA, USA) was utilized to detect protein signal via the LI-COR imaging system or the BioRad Chemidoc system. The following antibodies were used:, ATF2 (Cell Signaling, Frankfurt, Germany; #35031; dilution: 1:1000), ATF2 (Santa Cruz Biotechnology, Dallas, TX, USA; #sc-187; dilution: 1:500), ATF2 (Santa Cruz Biotechnology, Dallas, TX, USA; #sc-242(F2BR1); dilution 1:70), GAPDH (Santa Cruz Biotechnology, Dallas, TX, USA; #365062; dilution: 1:10,000), GAPDH (Santa Cruz Biotechnology, Dallas, TX, USA; #sc-32233; dilution: 1:20,000), H3 (Abcam, Berlin, Germany; #ab1791; dilution 1:25,000), p-ATF2 (Cell Signaling, Frankfurt, Germany; #9225; dilution: 1:1000), p-ATF2 (Thermo Fisher Scientific, Waltham, MA, USA; #05891; dilution: 1:2000), p-p38 (Cell Signaling, Frankfurt, Germany; #4511; dilution: 1:800), p-p38 (Cell Signaling, Frankfurt, Germany; #9211; dilution: 1:400), p38 (Cell Signaling, Frankfurt, Germany; #9212; dilution: 1:1000), p38alpha (Santa Cruz Biotechnology, Dallas, TX, USA; #sc-728; dilution: 1:500), Ucp1 (Abcam, Berlin, Germany; #ab23841; dilution: 1:2000), Ucp1 (Abcam; #ab10983; dilution: 1:6000), p-PKC (Cell Signaling, Frankfurt, Germany; #2060; dilution: 1:2000), ERK1/2 (Cell Signaling, Frankfurt, Germany; #9102; dilution: 1:2000), LC3A/B (Cell Signaling, Frankfurt, Germany; #4108; dilution: 1:2000), ATG7 (Cell Signaling, Frankfurt, Germany; #8558; dilution: 1:2000), alpha Tubulin (Santa Cruz Biotechnology, Dallas, TX, USA; #sc-8035; dilution: 1:1000), p-p44/42 MAPK (ERK1/2) (Cell Signaling, Frankfurt, Germany; #4511; dilution 1:1000). Images of uncropped blots are provided in the Source data file.

### Chromatin immunoprecipitation (ChIP) and real-time qPCR

ChIP-qPCR of mouse brown adipose tissue was performed with sonicated nuclear extract prepared from formaldehyde-cross-linked tissue, according to Mir et al.^25^, using two different ATF2 antibodies ATF2 (Bethyl Laboratories, Hamburg, Germany; #A301-649A; dilution: 1:300) or rabbit IgG (Cell Signaling, Frankfurt, Germany, #2729S) and two different p62 antibodies (Proteintech, Machester, UK; #18420-1AP; dilution 1:100) and p62 (Abcam, Berlin, Germany; #ab101266; dilution: 1:500). Immunoprecipitated DNA was decrosslinked, purified and used for quantitative real-time PCR. Primer sequences used are the following: *Foxl2* forward 5ʹ-GCTGGCAGAATAGCATCCG-3ʹ, *Foxl2* reverse 5ʹ-TGATGAAGCACTCGTTGAGGC-3ʹ, *Jun* forward 5ʹ-CGCAGCGGAGCATTACCTC-3ʹ, *Jun* reverse 5ʹ-GCCTGAGCTCAACACTTATCTG-3ʹ, *Fos* forward 5ʹ-GCTGAGGCGCCTACTACTCC-3ʹ, *Fos* reverse 5ʹ-TCATGGTCGAAGTTTGGGGAAA-3ʹ, *Atf3* forward 5ʹ-TTAGCCGATTGGCTCCACTG-3ʹ, *Atf3* reverse 5ʹ-CGTCAGCCTGGGATTGGTAA-3ʹ, *Ucp1* (CRE2) forward 5ʹ-AGTGAAGCTTGCTGTCACTC-3ʹ, *Ucp1* (CRE2) reverse 5ʹ-GTCTGAGGAAAGGGTTGACC-3ʹ, *Ucp1* (CRE4) forward 5ʹ-GAGTGACGCGCGGCTGGG-3ʹ, *Ucp1* (CRE4) reverse 5ʹ-GGGCTAGGTAGTGCCAGT-3ʹ, *Pgc-1α* forward 5ʹ-ACTATAGGGCACGCGTGGTCGACG-3ʹ, *Pgc-1α* reverse 5ʹ-ACACAGAGCACACACTCATGCAGG-3ʹ.

### Luciferase assay

ATF2 transcriptional activity was monitored in vitro using a reporter construct consisting of four tandem copies of the CRE-binding sequence TGACGTCA linked to luciferase. HEK293T cells (ATCC, Manassas, VA, USA) were transiently transfected with the following plasmids using Lipofectamine 2000 according to manufacturer’s (Thermo Fisher, Waltham, MA, USA): pCRE-Luc, pRL-TK, pcDNA3-Flag MKK6(K82A) (Addgene, Watertown, MA, USA; #13519)^26^, pcDNA3.1-C-DYK-p62wt, pcDNA3.1-C-DYK-p62^∆69-251^, pcDNA3.1. The ATF2 reporter (pCRE-Luc) was a kind gift from Takayuki Akimoto.

Transfection was performed in collagen A-coated 96-well plates using 0.25 µg plasmid DNA and 0.875 µl Lipofectamine 2000 reagent in a volume of 25 µl Optimem media. To warrant equal DNA uptake of the cells, the plasmid DNA concentration was adjusted with an empty vector (pcDNA3.1). CRE-binding activity reporter assay was performed using the Dual-Glo luciferase assay (Promega, Madison, WI, USA), which allows normalization to transfection efficiency by simultaneous measurement of firefly and renilla (pRL-TK) luminescence. Therefore, the pRL-TK construct was routineously co-transfected to all samples. Luciferase assay was performed according to protocol and luminescence signal was measured using a PHERAstar plate reader.

### p62 Transfection studies

Primary brown adipocytes isolated from p62^−/−^ mice were transiently transfected with the following plasmids using Lipfectamine P3000 according to manufacturer’s protocol (Thermo Fisher, Waltham, MA, USA): pcDNA3.1 and pcDNA3.1-C-DYK-p62wt. Transfection was performed on Day 4 of differentiation in 6-well plates using 4 µg plasmid DNA and 12 µl Lipofectamine reagent in 250 µl OptiMem medium. Cells were harvested in RIPA buffer for western blot analysis on Day 6 of differentiation.

### Statistics

Statistical analyses were performed using statistical tools in GraphPad Prism8 (version 8.3.0). Differences between groups were assessed by Student’s two-sided two-tailed *t*-test, one-way ANOVA, or two-way ANOVA with time and genotype as co-variants followed by Bonferroni’s post hoc multiple comparison testing for individual time points. All results are given as mean ± SEM. *P* < 0.05 was considered statistically significant. Differences in energy expenditure were calculated using ANCOVA with body weight as covariate using SPSS (version 24). For animal studies, sample sizes were calculated based on a power analysis assuming that a ≥5 g difference in body weight between genotypes can be assessed with a power of ≥75% when using a two-sided statistical test under the assumption of a standard deviation of 3.5 and an alpha level of 0.05.

### Reporting summary

Further information on research design is available in the Nature Research Reporting Summary linked to this article.

## Supplementary information

Supplementary Information

Reporting Summary

## Data Availability

The data that support the findings of this study are available from the corresponding author upon reasonable request. The source data underlying Figs. 1a–j, 2a–i, 3a–e, 4a, c–j, 5a, b, d, 7a–c, Supplementary Figs. 1b–e, h, i, 2a–f, 3b–d, 4a–f, 5b–f, h–l, 6a–h, 7a–I, 8a, b, 9a, b, 10a, b are provided as a Source data file.
