# Supplementary material for: The scaffold protein p62 regulates adaptive thermogenesis through ATF2 nuclear target activation
Source: Nat Commun. 2020 May 8;11:2306. doi: 10.1038/s41467-020-16230-8 (PMC7211001; doi:10.1038/s41467-020-16230-8)
Supplement: Supplementary file 1 — Supplementary Information [file 41467_2020_16230_MOESM1_ESM.pdf]

Supplementary Figures

The scaffold protein p62 regulates adaptive thermogenesis through ATF2 nuclear target activation

Katrin Fischer[1,*], Anna Fenzl[1,*], Dianxin Liu [2], Kenneth A. Dyar[1], Maximilian Kleinert[1,3,4], Markus Brielmeier[5], Christoffer Clemmensen[1,6] , Anna Fedl[1], Brian Finan[1,7] , Andre Gessner[8], Martin Jastroch[1,9], Jianfeng Huang[10], Susanne Keipert[1,9], Martin Klingenspor[11,12], Jens C. Brüning[13], Manfred Kneilling[14,15], Florian C. Maier[14], Ahmed E. Othman[16], Bernd J. Pichler[14], Ines Pramme-Steinwachs[1], Stephan Sachs[1], Angelika Scheideler[5], Wolfgang M. Thaiss[14,16], Henriette Uhlenhaut[1], Siegfried Ussar[1], Stephen C. Woods[17], Julia Zorn[4], Kerstin Stemmer[18], Sheila Collins[2,] Maria Diaz-Meco[10], Jorge Moscat[10], Matthias H. Tschöp[1,3], Timo D. Müller[1,19#]

[1]Institute for Diabetes and Obesity, Helmholtz Diabetes Center (HDC), Helmholtz Zentrum München and German National Diabetes Center (DZD), 85764 Neuherberg, Germany. [2]Division of Cardiovascular Medicine, Vanderbilt University Medical Center, 37232 Nashville, TN, USA. [3]Division [3]Division of Metabolic Diseases, Department of Medicine, Technische Universität München, 80333 Munich, Germany. [4]Section of Molecular Physiology, Department of Nutrition, Exercise and Sports, Faculty of Science, University of Copenhagen, 2200, Copenhagen, Denmark. [5]Research Unit Comparative Medicine, Helmholtz Zentrum München, German Research Center for Environmental Health GmbH, Neuherberg, Germany.[6]Novo Nordisk Foundation Center for Basic Metabolic Research, Faculty of Health and Medical Sciences, University of Copenhagen, Blegdamsvej 3B, 2200, Copenhagen, Denmark. [7]Novo Nordisk Research Center, Indianapolis, Indiana. [8]Institute of Clinical Microbiology and Hygiene, University Hospital Regensburg, Germany. [9]Department of Molecular Biosciences, The Wenner-Gren Institute, The Arrhenius Laboratories F3, Stockholm University, SE-106 91 Stockholm, Sweden.[10] Cancer Metabolism and Signaling Networks Program, Sanford Burnham Prebys Medical Discovery Institute, La Jolla, CA92037, USA. [11] Chair of Molecular Nutritional Medicine, Technical University of Munich, TUM School of Life Sciences Weihenstephan, Gregor-Mendel-Strasse 2, D-85354 Freising, Germany. [12]EKFZ – Else-Kröner Fresenius Center for Nutritional Medicine, Technical University of Munich, Gregor-Mendel-Strasse 2, D-85354 Freising, Germany. [13]Department of Neuronal Control of Metabolism, Max Planck Institute for Metabolism Research, Gleueler Strasse 50, 50931 Cologne, Germany; Policlinic for Endocrinology, Diabetes and Preventive Medicine (PEDP), University Hospital Cologne, Kerpener Strasse 26, 50924 Cologne, Germany; Excellence Cluster on Cellular Stress Responses in Aging-Associated Diseases (CECAD) and Center for Molecular Medicine Cologne (CMMC), University of Cologne, Joseph-Stelzmann-Strasse 26, 50931 Cologne, Germany.[14]Werner Siemens Imaging Center, Department of Preclinical Imaging and Radiopharmacy, Eberhard Karls University Tübingen, Germany. [15]Department of Dermatology, Eberhard Karls University Tübingen, 72076 Tübingen, Germany. [16]Department of Diagnostic and Interventional Radiology, Eberhard Karls University Hospital Tübingen, Hoppe-Seyler-Straße 3, 72076 Tübingen, Germany. [17]Metabolic Disease Institute, Department of Psychiatry and Behavioral Neuroscience, University of Cincinnati, OH45237 Cincinnati, USA. [18]Department of Biology, University of Konstanz, Konstanz Germany. [19]Institute of Experimental and Clinical Pharmacology and Pharmacogenomics, Department of Pharmacology, Experimental Therapy and Toxicology, Eberhard Karls University Hospitals and Clinics, 72076 Tübingen, Germany.

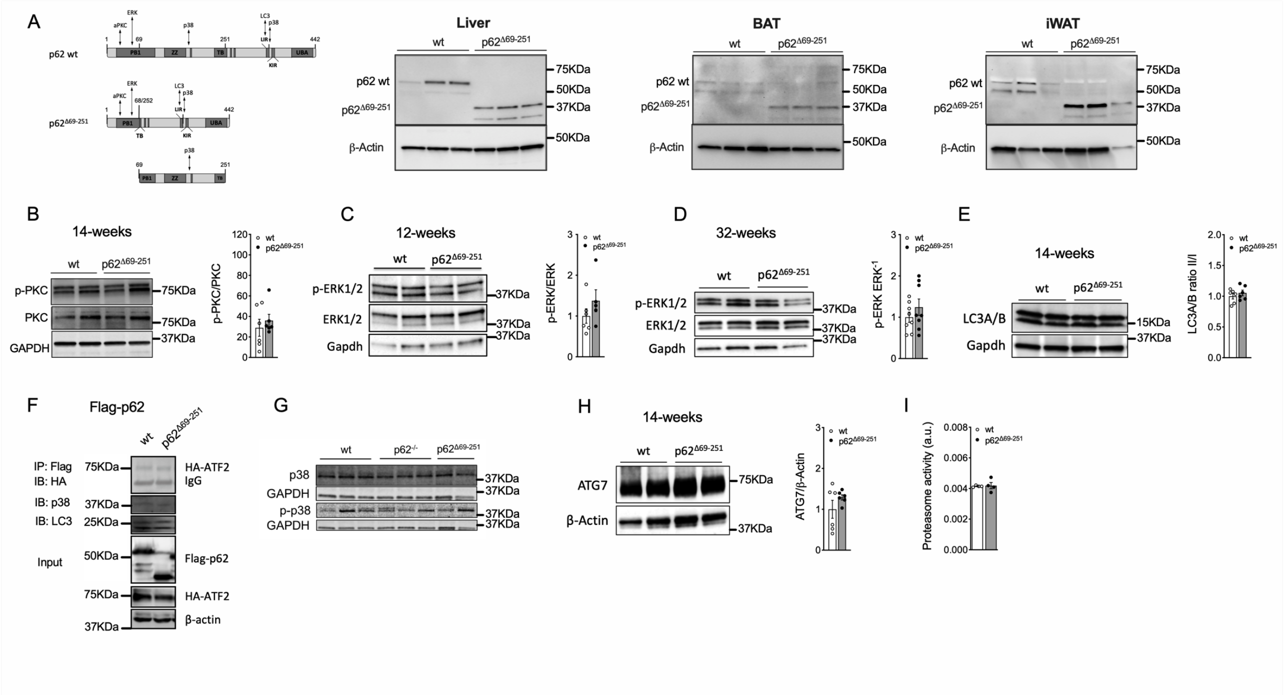

Supplementary Figure 1: Protein quantification of p62 interacting molecules in p62$^{\Delta 69-251}$ mice. Structural domains in p62 wt and p62$^{\Delta 69-251}$ mice and protein level of p62 in liver, BAT and iWAT of 11 week old male wt (n=3) or p62$^{\Delta 69-251}$ mice (n=3) (A). Protein level of phosphorylated and total PKC in liver of lean 14 wk old male mice (B). Protein levels of total and phosphorylated ERK1/2 in iWAT of 12 wk old lean (C) or 32 wk old obese (D) male wt or p62$^{\Delta 69-251}$ mice. LC3A/B protein levels in liver of 14 wk old male wt or p62$^{\Delta 69-251}$ mice (E). Co-Immunoprecipitation in HEK293FT cells co-transfected with either Flag-tagged- p62 wt or p62$^{\Delta 69-251}$ and HA-ATF2 (F). Total and phosphorylated p38 protein levels in BAT of lean 8-10 wk old male wt, p62$^{-/-}$ or p62$^{\Delta 69-251}$ mice (G). ATG7 protein quantification in liver of 14 wk old male wt or p62$^{\Delta 69-251}$ mice (H). Proteasome assay in BAT primary cells harvested from wt or p62$^{\Delta 69-251}$ mice (I). Panel B is a representative blot of n=2/2 (out of n=6/6) mice. Panel C is a representative blot of n=2/2 (out of n=5/6) mice. Panel D is a representative blot of n=2/2 (out of n=8/8) mice. Panel E is a representative blot of n=2/2 (out of n=6/6) mice. Panel F is a representative example of three independently performed studies, each yielding similar results. Panel G comprises n=3/3/2 mice and has been independently confirmed in another n=3/3/2 mice. Panel H is a representative blot of n=2/2 (out of n=6/6) mice. Panel I comprise n=4/4 technical replicates and is a representative example of three independently performed studies. Full uncropped western blots are shown in the data source file. Data have been analyzed using 2-sided 2-tailed t-test. Data represent mean ± SEM. *$p < 0.05$; **$p < 0.01$; ***$p < 0.001$. Exact p-values are (B) p=0.509, (C) p=0.262, (D) p=0.315, (E) p=0.426, (H)p=0.232 and (I) p=0.844. Abbreviations: PKC protein kinase C, ERK1/2 mitogen-activated protein kinase 1/2, LC3A/B microtubule-associated protein 1 light chain 3 alpha/beta, ATG7 Autophagy related 7.

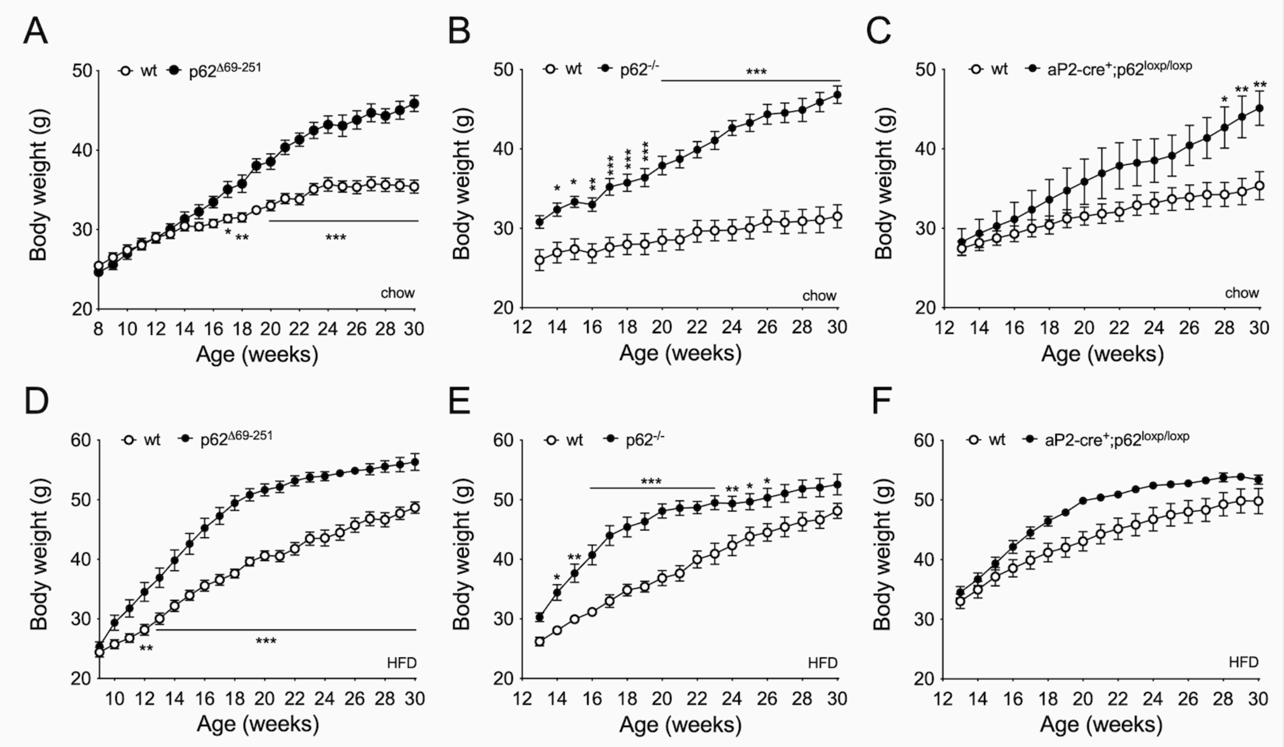

Supplementary Figure 2. Side by side comparison of body weight in global p62$^{-/-}$, adipocyte-specific p62$^{-/-}$ and p62$^{\Delta 69\text{-}251}$ mice. Body weight of male p62$^{\Delta 69\text{-}251}$, global p62$^{-/-}$ or adipose tissue-specific p62$^{-/-}$ (aP2-cre^{+};p62$^{loxp/loxp}$) mice held on either a standard chow (A-C) or high-fat diet (HFD) (D-F). Sample sizes are (A) n=9/7 mice, (B) n=8/8 mice, (C) n=13/7 mice, (D), n=7/9 mice, (E) n=8/8 mice, (F) n=12/7 mice. Data have been analyzed using 2-way ANOVA with time and genotype as co-variant followed by Bonferroni post-hoc multiple comparison testing for differences at individual time points. Data represent mean ± SEM. **p* < 0.05; ***p* < 0.01; ****p* < 0.001. Exact p-values are (A) 17 wks p=0.216, 18 wks p=0.007, 19-30 weeks p<0.0001; (B) 14 wks p=0.0354, 15 wks p=0.0123, 16 wks p=0.0003, 17 wks p=0.0002, 18 - 30 wks p<00001; (C) 28 wks p=0.0178, 29 wks p=0.0045, 30 wks p=0.0026; (D) 12 wks p=0.0029, 13 wks p=0.0008, 14 wks p=0.0001, 15-29 wks p<0.0001, 30 wks p=0.0001; (E) 14 wks p=0.0175, 15 wks p=0.0012, 16 – 21 wks p<0.0001, 22 wks p=0.0001, 23 wks p=0.0002, 24 wks p=0.0048, 25 wks p=0.0439, 26 wks p=0.0430.

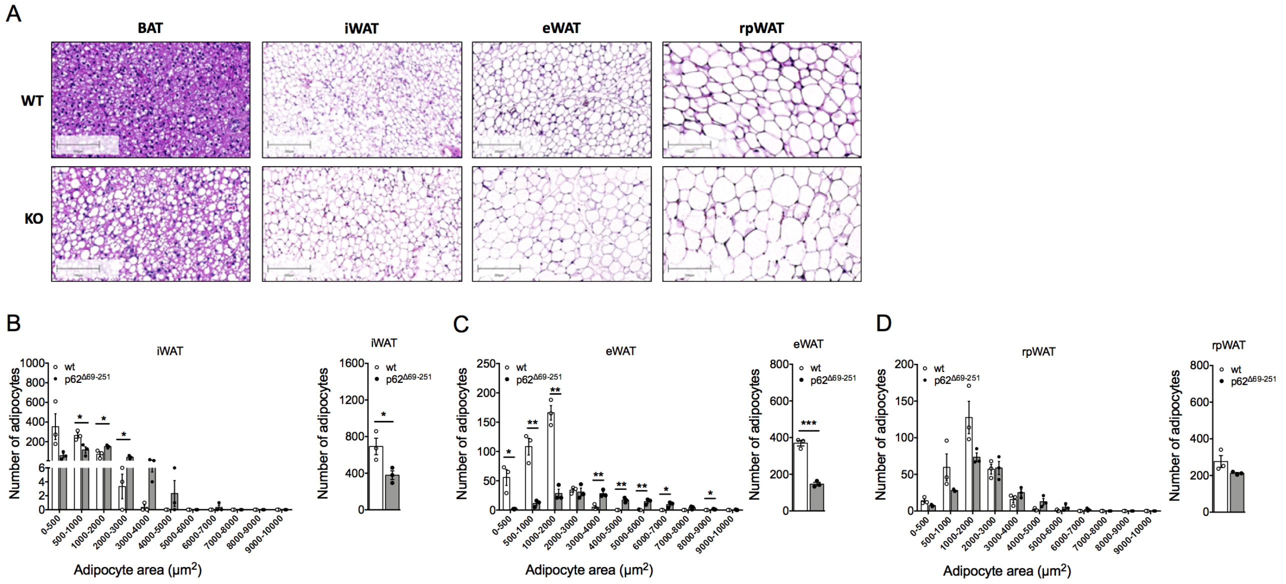

Supplementary Figure 3. Adipocyte morphology in adipose depots of lean wt or p62$^{\Delta 69\text{-}251}$ mice. HE staining of BAT, iWAT, eWAT and rpWAT from 14 wk old male C57Bl/6J wt or p62$^{\Delta 69\text{-}251}$ mice (A). Scale bar = 200 μm. Number of adipocytes clustered per size and total number of adipocytes quantified per image in iWAT (B), eWAT (C) and rpWAT (D) of 14 wk old male C57Bl/6J wt or p62$^{\Delta 69\text{-}251}$ mice. Panel A is a representative example of n=3 mice analyzed from each genotype. Quantification in B-D is based on n=3 mice per genotype. Data have been analyzed using 2-sided 2-tailed t-test. Data represent mean ± SEM. *$p < 0.05$; **$p < 0.01$; ***$p < 0.001$. Exact p-values are (B) 0 – 500 p=0.0911, 500 – 1000 p= 0.0256, 1000 – 2000 p=0.0355, 2000 – 3000 p=0.0201, 3000 – 4000 p=0.085, 4000 – 5000 p=0.277; separate bar graph p=0.037; (C) 0 – 500 p=0.0162, 500 – 1000 p= 0.00272, 1000 – 2000 p=0.000673, 2000 – 3000 p=0.681, 3000 – 4000 p=0.00599, 4000 – 5000 p=0.00181; 5000 – 6000 p=0.00701, 6000 – 7000 p=0.0268, 7000 – 8000 p=0.0589, 8000 – 9000 p=0.0161, separate bar graph p=0.000237; (D) 0 – 500 p=0.0668, 500 – 1000 p=0.1600, 1000 – 2000 p=0.077, 2000 – 3000 p=0.888, 3000 – 4000 p=0.192, 4000 – 5000 p=0.0777; separate bar graph p=0.037; (C) 0 – 500 p=0.0162, 500 – 1000 p= 0.00272, 1000 – 2000 p=0.000673, 2000 – 3000 p=0.681, 3000 – 4000 p=0.00599, 4000 – 5000 p=0.00181; 5000 – 6000 p=0.1198. Abbreviations: iWAT inguinal white adipose tissue, eWAT epididymal white adipose tissue, rpWAT retroperitoneal white adipose tissue.

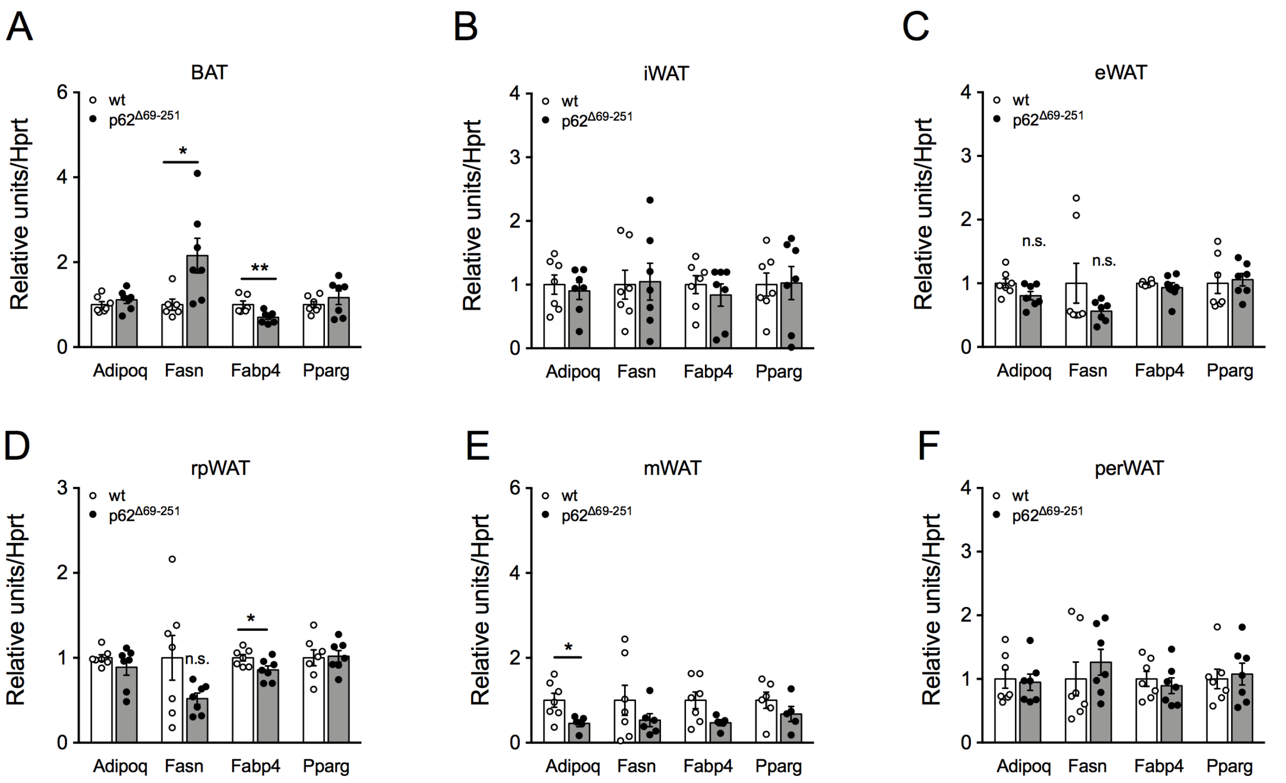

Supplementary Figure 4. Adipogenic gene expression profiling in lean wt and $p62^{\Delta69-251}$ mice. Gene expression of adipogenic markers (*Adipoq*, *Fasn*, *Fabp4* and *Pparg)* in BAT (A), iWAT (B), eWAT (C), rpWAT (D), mWAT (E) and perWAT (F) of 14 wk old male C57Bl/6J wt or $p62^{\Delta69-251}$ mice. Sample sizes are (A) *Adipoq* n=7/7 mice, *Fasn* n=6/7 mice, *Fabp4* n=6/7 mice, *Pparg* n=7/7 mice, (B) *Adipoq* n=7/7 mice, *Fasn* n=7/7 mice, *Fabp4* n=7/7 mice, *Pparg* n=7/7 mice, (C) *Adipoq* n=7/7 mice, *Fasn* n=7/7 mice, *Fabp4* n=6/7 mice, *Pparg* n=7/7 mice, (D) *Adipoq* n=7/7 mice, *Fasn* n=7/7 mice, *Fabp4* n=7/7 mice, *Pparg* n=7/7 mice, (E) Adipoq n=7/5 mice, Fasn n=7/5 mice, Fabp4 n=7/5 mice, Pparg n=6/5 mice, (F) *Adipoq* n=7/7 mice, *Fasn* n=7/7 mice, *Fabp4* n=7/7, *Pparg* n=7/7 mice. Data represent mean ± SEM. Data have been analyzed using 2-sided 2-tailed t-test **p* < 0.05; ***p* < 0.01; ****p* < 0.001. Exact p-values are (A) *Adipoq* p=0.335, *Fasn* p=0.029, *Fabp4* p=0.0096, *Pparg* p=0.268; (B) *Adipoq* p=0.632, *Fasn* p=0.901, *Fabp4* p=0.483, *Pparg* p=0.938; (C) *Adipoq* p=0.068, *Fasn* p=0.194, *Fabp4* p=0.430, *Pparg* p=0.764; (D) *Adipoq* p=0.277, *Fasn* p=0.101, Fabp4 p=0.0382, Pparg p=0.881; (E) Adipoq p=0.0263, Fasn p=0.188, Fabp4 p=0.0541, *Pparg* p=0.250; (F) *Adipoq* p=0.792, *Fasn* p=0.448, *Fabp4* p=0.540, *Pparg* p=0.745. Abbreviations: *Adipoq* adiponectin, *Fasn* fatty acid synthase, *Fabp4* fatty acid binding protein 4, *Pparg* Peroxisome proliferator activated receptor gamma.

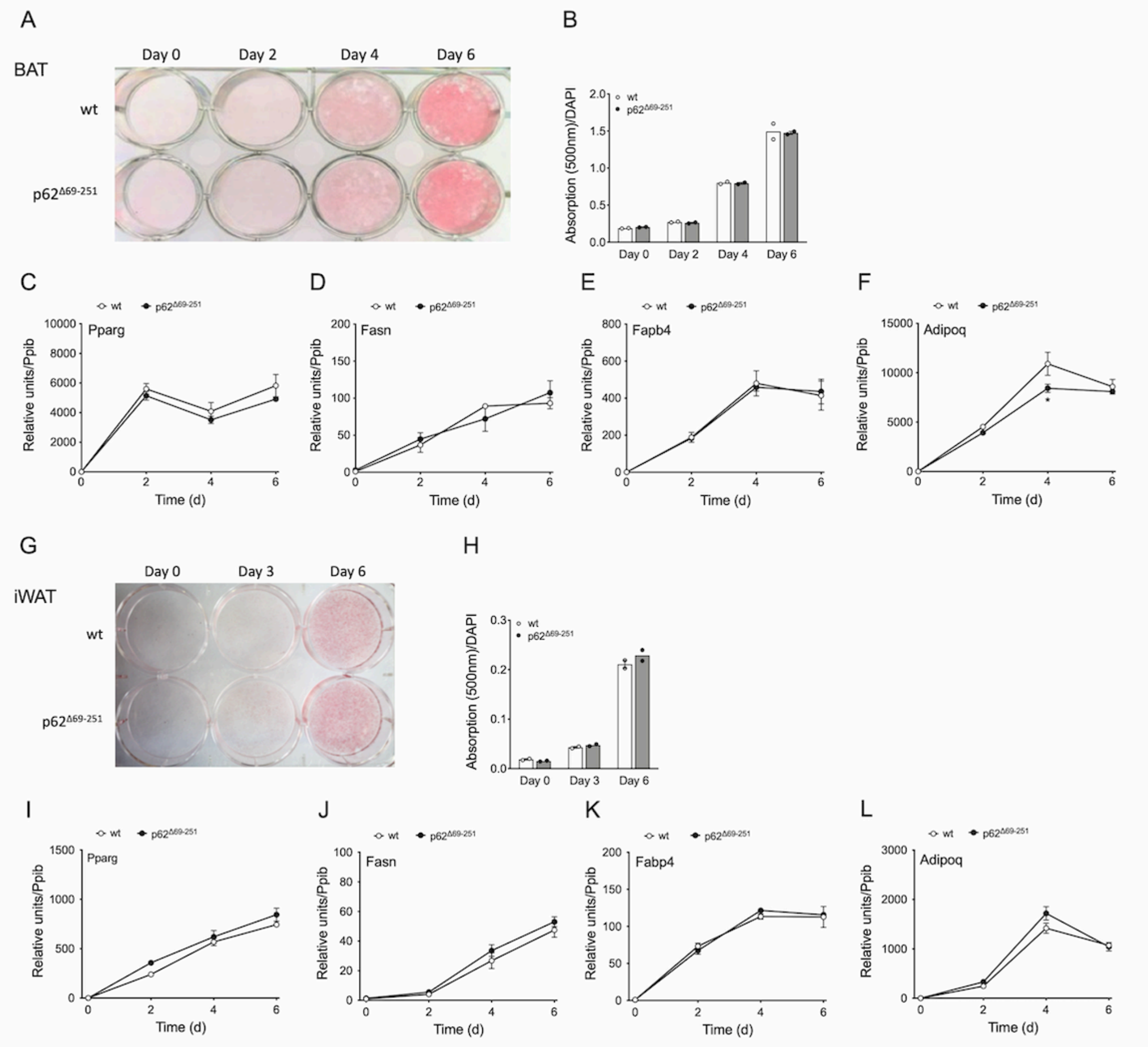

Supplementary Figure 5. Adipogenic marker in white and brown primary adipocytes of wt or p62$^{\Delta 69\text{-}251}$ mice during differentiation. Oil-Red-O staining (**A**) and lipid quantification via elution of Oil-Red-O dye (B) of wt and p62$^{\Delta 69\text{-}251}$ BAT primary cells at day 0, 2, 4 or 6 of adipocyte differentiation. Gene expression of *Pparg, Fasn*, *Fabp4* and *Adipoq* in wt and p62$^{\Delta 69\text{-}251}$ BAT primary cells (C-F). Oil-Red-O staining (G) and lipid quantification via elution of Oil-Red-O dye (H) of wt and p62$^{\Delta 69\text{-}251}$ iWAT primary cells at day 0, 3 or 6 of adipocyte differentiation. Gene expression of *Pparg, Fasn*, *Fabp4* and *Adipoq* in wt or p62$^{\Delta 69\text{-}251}$ iWAT primary cells (I-L). (B,G) Quantification of lipids from Oil-Red-O dye elution was done in n=2 technical replicates each well. (C-F) BAT primary cells were harvested from 6-8 wk old male p62 WT or p62$^{\Delta 69\text{-}251}$ mice and plated with equal number of cells in n=3-4 wells each genotype. Cells were then individually differentiated into mature brown adipocytes followed by measurement of target gene expression with n=2 technical replicates each well. Sample sizes are (C) n=4/4 individually differentiated wells, (D), n=3/4 individually differentiated wells, (E) n=4/4 individually differentiated wells, (F) n=4/4 individually differentiated wells. Results in C-F are representative of two independently performed studies, each yielding similar results. (I-L) iWAT primary cells were harvested from 6-8 wk old male p62 WT or p62$^{\Delta 69\text{-}251}$ mice and plated with equal number of cells in n=4 wells each genotype. Cells were then individually differentiated into mature white adipocytes followed by measurement of target gene expression with n=2 technical replicates each well. Sample sizes are (I) n=4/4 individually differentiated wells, (J), n=4/4 individually differentiated wells, (K) n=4/4 individually differentiated wells (L) n=4/4 individually differentiated wells. Results in I-L are representative of two independently performed studies, each yielding similar results. Data in C-F and I-L have been analyzed using 2-way ANOVA using time and genotype as

co-variants and using Bonferroni post-hoc multiple comparison for testing of differences at individual time points. Data represent mean ± SEM. *p < 0.05; **p < 0.01; ***p < 0.001. Exact p-value is (F) d 4 p=0.0137.

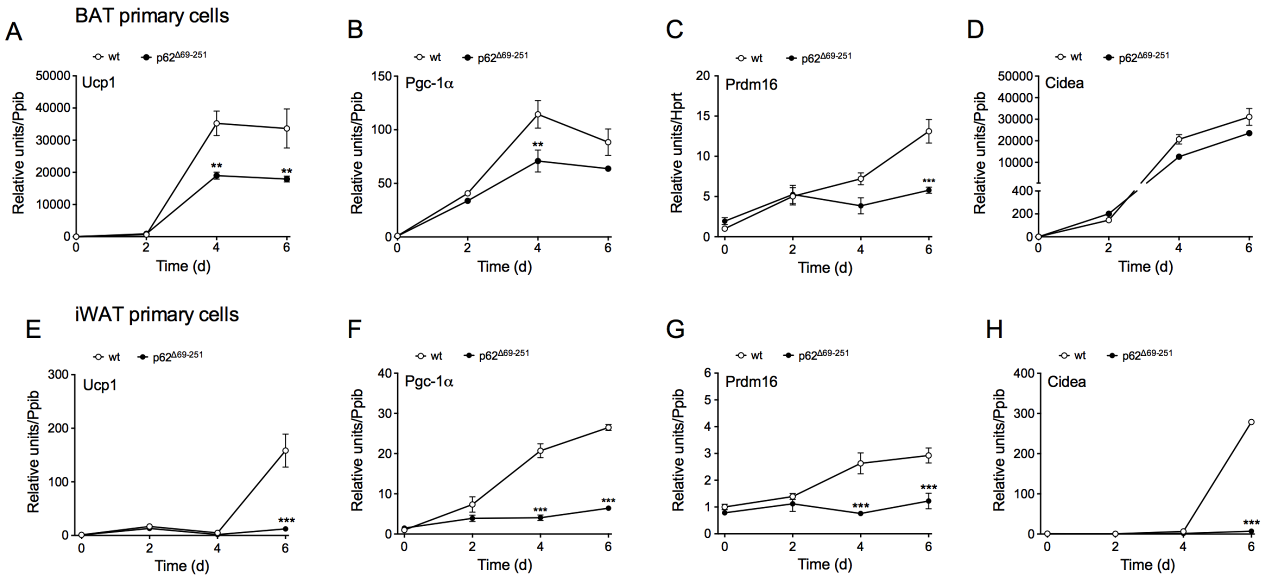

Supplementary Figure 6. Thermogenic gene expression in white and brown primary adipocytes of wt or p62$^{\Delta69\text{-}251}$ mice. Gene expression of markers indicative of adaptive thermogenesis and browning (*Ucp1*, *Pgc-1α*, *Prdm16*, *Cidea*) in BAT (**A**-**D**) and iWAT (**E**-**H**) primary cells of male wt or p62$^{\Delta69\text{-}251}$ mice during adipocyte differentiation. (A-D) BAT primary cells were harvested from 6-8 wk old male p62 WT or p62$^{\Delta69\text{-}251}$ mice and plated with equal number of cells in n=4 wells each genotype. Cells were then individually differentiated into mature brown adipocytes followed by measurement of target gene expression with n=2 technical replicates each well. Sample sizes are (A) n=4/4 individually differentiated wells, (B) n=4/4 individually differentiated wells, (C) n=4/4 individually differentiated wells, (D) 4/4 individually differentiated wells. Results in A-D are representative of two independently performed studies, each yielding similar results. (E-H) iWAT primary cells were harvested from 6-8 wk old male p62 WT or p62$^{\Delta69\text{-}251}$ mice and plated with equal number of cells in n=3 wells each genotype. Cells were then individually differentiated into mature white adipocytes followed by measurement of target gene expression with n=2 technical replicates each well. Sample sizes are (E) n=3/3 individually differentiated wells, (F) n=3/3 individually differentiated wells, (G) n=3/3 individually differentiated wells, (H) n=3/3 individually differentiated wells. Results in E-H are representative of two independently performed studies, each yielding similar results. Data represent mean ± SEM. Data in A-H have been analyzed using 2-way ANOVA using time and genotype as co-variants and using Bonferroni post-hoc multiple comparison for testing of differences at individual time points. *p < 0.05; **p < 0.01; ***p < 0.001. Exact p-values are (A) d4 p=0.0045, d6 p=00061; (B) d4 p=0.0012; (C) d6 p<0.0001; (E) d6 p<0.0001; (F) d4 p<0.0001, d6 d4 p<0.0001; (G) d4 p=0.0001; d6 p=0.0004; (H) d6 p<0.0001.

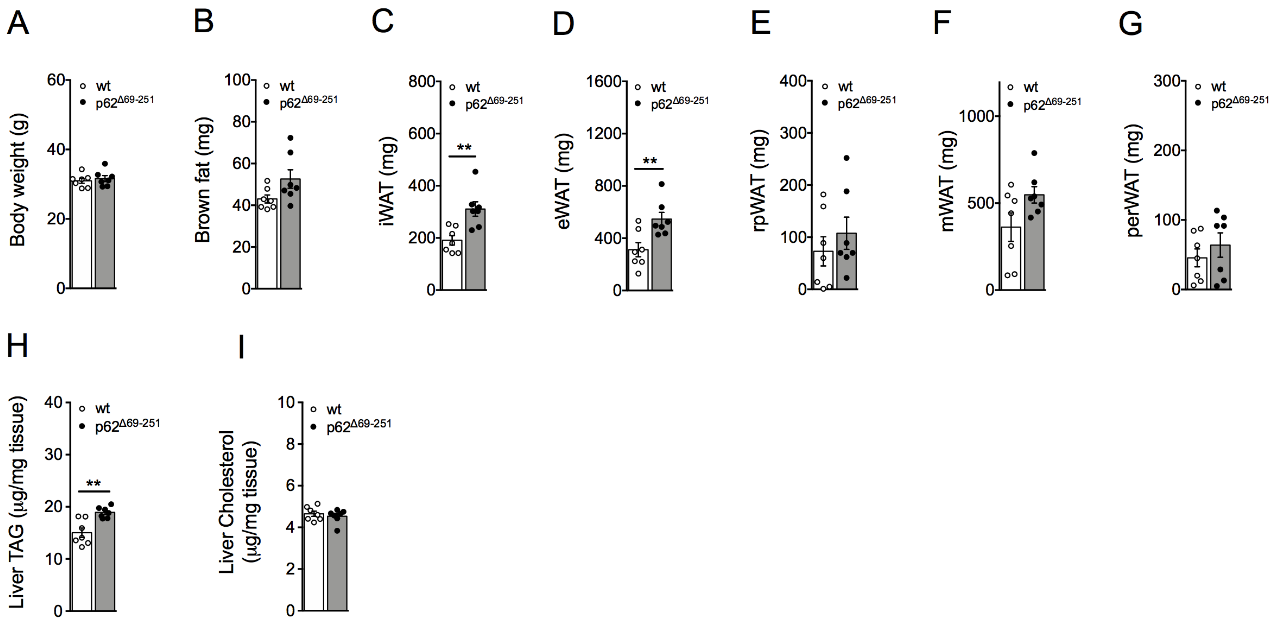

Supplementary Figure 7. Quantification of fat depots in lean 14 wk old wt or p62$^{\Delta 69\text{-}251}$ mice. Body weight (A) and tissue wet weight of BAT (B), iWAT (C), eWAT (D), rpWAT (E), mWAT (F), perWAT (G) of 14 wk old male C57Bl/6J wt or p62$^{\Delta 69\text{-}251}$ mice. Hepatic levels of triglycerides (H) and cholesterol (I) in 14 wk old male C57Bl/6J wt or p62$^{\Delta 69\text{-}251}$ mice. Sample sizes are (A-I) n=7/7 mice. Data represent mean ± SEM. Data have been analyzed using 2-sided 2-tailed t-test. **P* < 0.05; ***P* < 0.01; ****P* < 0.001. Exact p-values are (A) p=0.594, (B) p=0.071, (C) p=0.00347, (D) p=0.00892, (E) p=0.4209, (F) p=0.0734, (G) p=0.4139, (H) p=0.0021, (I) p=0.5214.

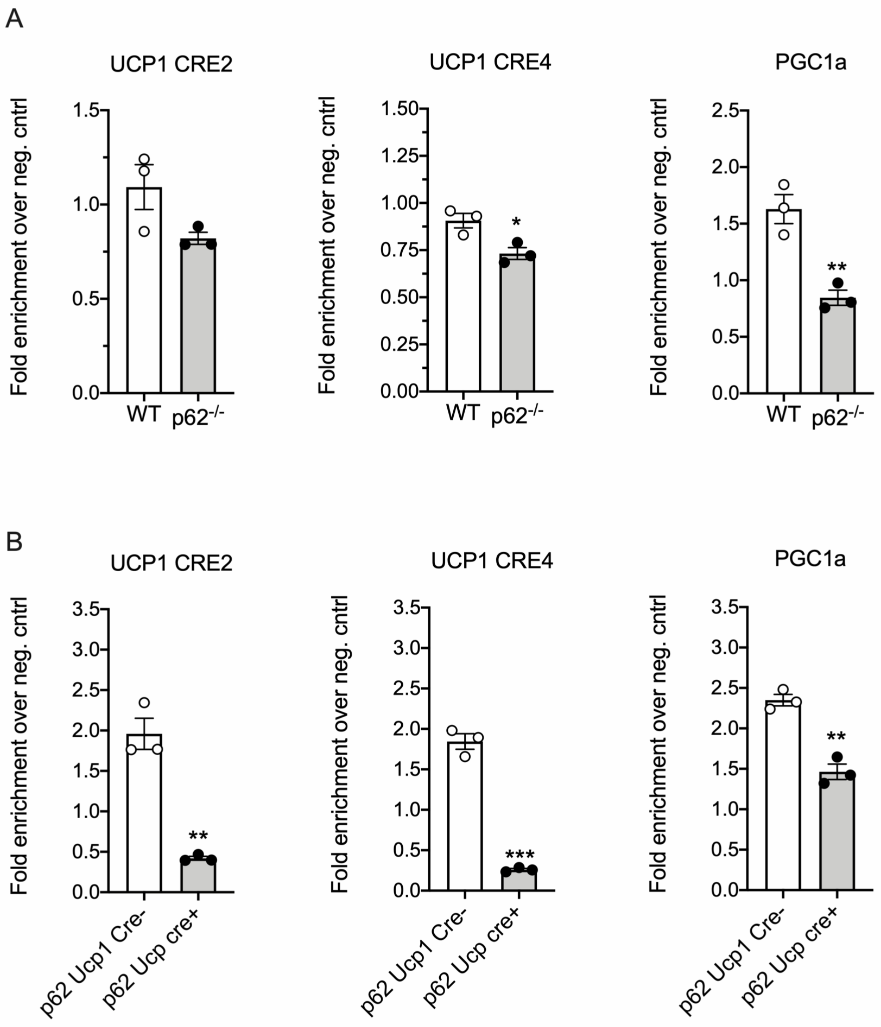

Supplementary Figure 8. Impaired chromatin binding of p62 in global p62$^{-/-}$ and BAT-specific p62$^{-/-}$ mice. Chromatin immunoprecipitation (ChIP) in BAT of wt and p62$^{-/-}$ mice (A) and BAT-specific p62$^{-/-}$ mice (B) using antibodies specific for p62. ChIP qPCR analysis of *Foxl2* (negative control), *Ucp1 (CRE2)*, *Ucp1 (CRE4)* and *Pgc-1α* (n=4 mice pooled each genotype, n=3 independently performed ChIP studies, each done with n=2 technical replicates) (A, B). Data represent mean ± SEM. Data have been analyzed using 2-sided 2-tailed t-test. *p < 0.05; **p < 0.01; ***p < 0.001. Exact p-values are (A) UCP1 CRE 2 p=0.0921, UCP1 CRE 4 p=0.0247, Pgc1a p=0.0056; (B) UCP1 CRE 2 p=0.00137, UCP1 CRE 4 p=0.000084, Pgc1a p=0.00174.

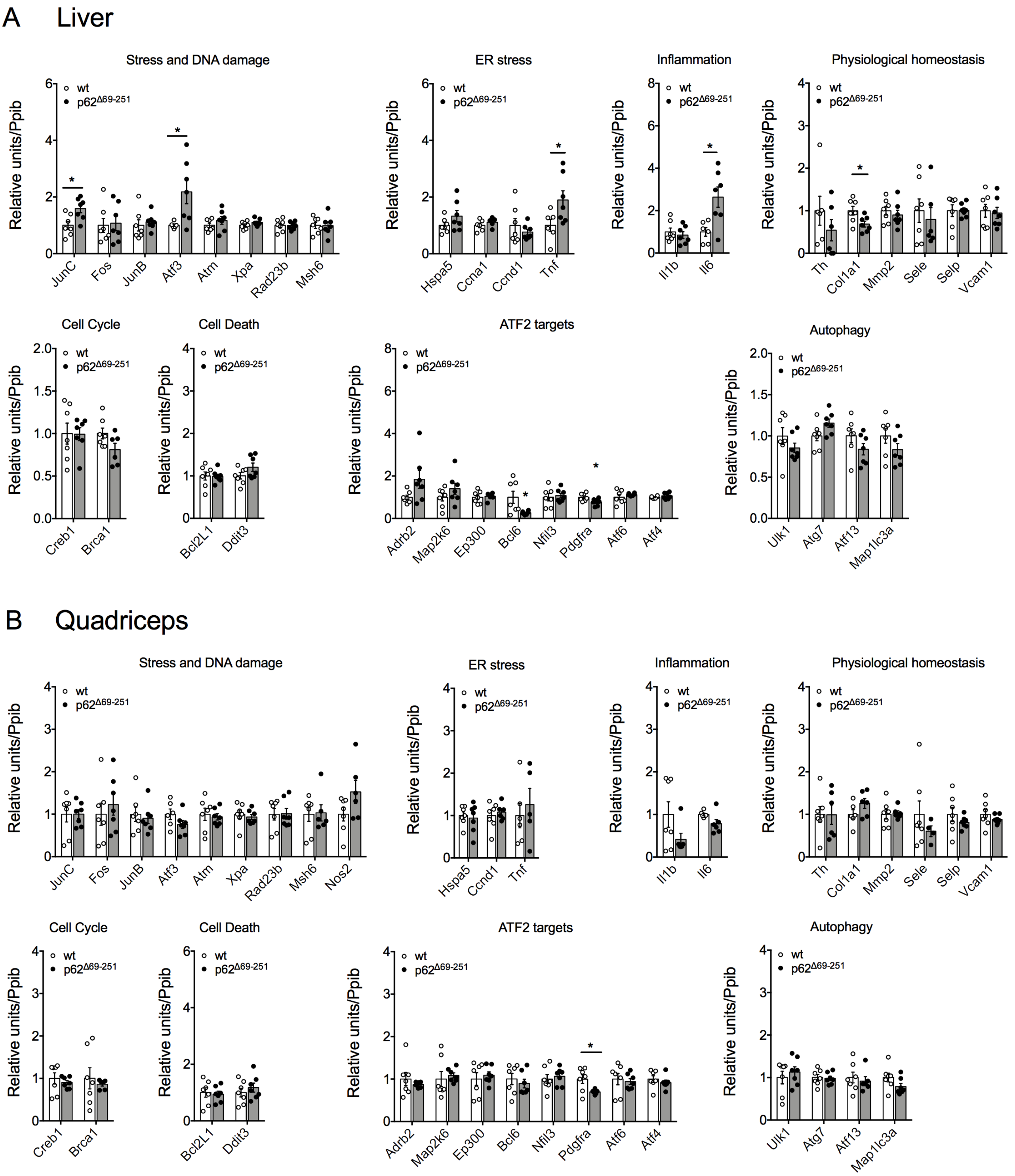

Supplementary Figure 9. Gene expression analysis of liver and muscle from lean wt or p62$^{\Delta69\text{-}251}$ mice. Analysis of known ATF2 targets implicated in stress and DNA damage, ER stress, inflammation, physiological homeostasis, cell cycle, cell death and autophagy in liver (A) and quadriceps (B) of 14 wk old male C57Bl/6J wt or p62$^{\Delta69\text{-}251}$ mice. Data have been analyzed using 2-sided 2-tailed t-test. Data represent mean ± SEM (n=6-7 mice per group). **p* < 0.05; ***p* < 0.01; ****p* < 0.001. Abbreviations: JunC jun proto-oncogene, Fos FBJ osteosarcoma oncogene, JunB JunB proto-oncogene, Atf3 activating transcription factor 3, Atm ATM serine/threonine kinase, Xpa XPA, DNA damage recognition and repair factor, Rad23b RAD23 homolog B, nucleotide excision repair protein, Msh6 mutS homolog 6, Nos2 nitric oxide synthase 2, Hsp5 FK506 binding protein 4, Ccnd1 cyclin D1, Tnf

tumor necrosis factor, Il1b interleukin 1 beta, Il6 interleukin 6, th tyrosine hydroxylase, Col1a1 collagen type I alpha 1, Mmp2 matrix metallopeptidase 2, Sele selectin E, Selp selectin P, Vcam1 vascular cell adhesion molecule 1, Creb1 cAMP responsive element binding protein 1, Brca1 BRCA1, DNA repair associated, Bcl2L1 BCL2 like 1, Ddit3 DNA damage inducible transcript 3, Adrb2 adrenoceptor beta 2, Map2k6 mitogen-activated protein kinase kinase 6, Ep300 E1A binding protein p300, Bcl6 B-cell CLL/lymphoma 6, Nfil3 nuclear factor, interleukin 3 regulated, Pdgfra platelet derived growth factor receptor alpha, Atf6 activating transcription factor 6, Atf4 activating transcription factor 4, Ulk1 unc-51 like autophagy activating kinase 1, Atg7 activating transcription factor 7, Atf13 activating transcription factor 13, Map1lc3a microtubule associated protein 1 light chain 3 alpha. The exact p-values are given in the Data Source File.

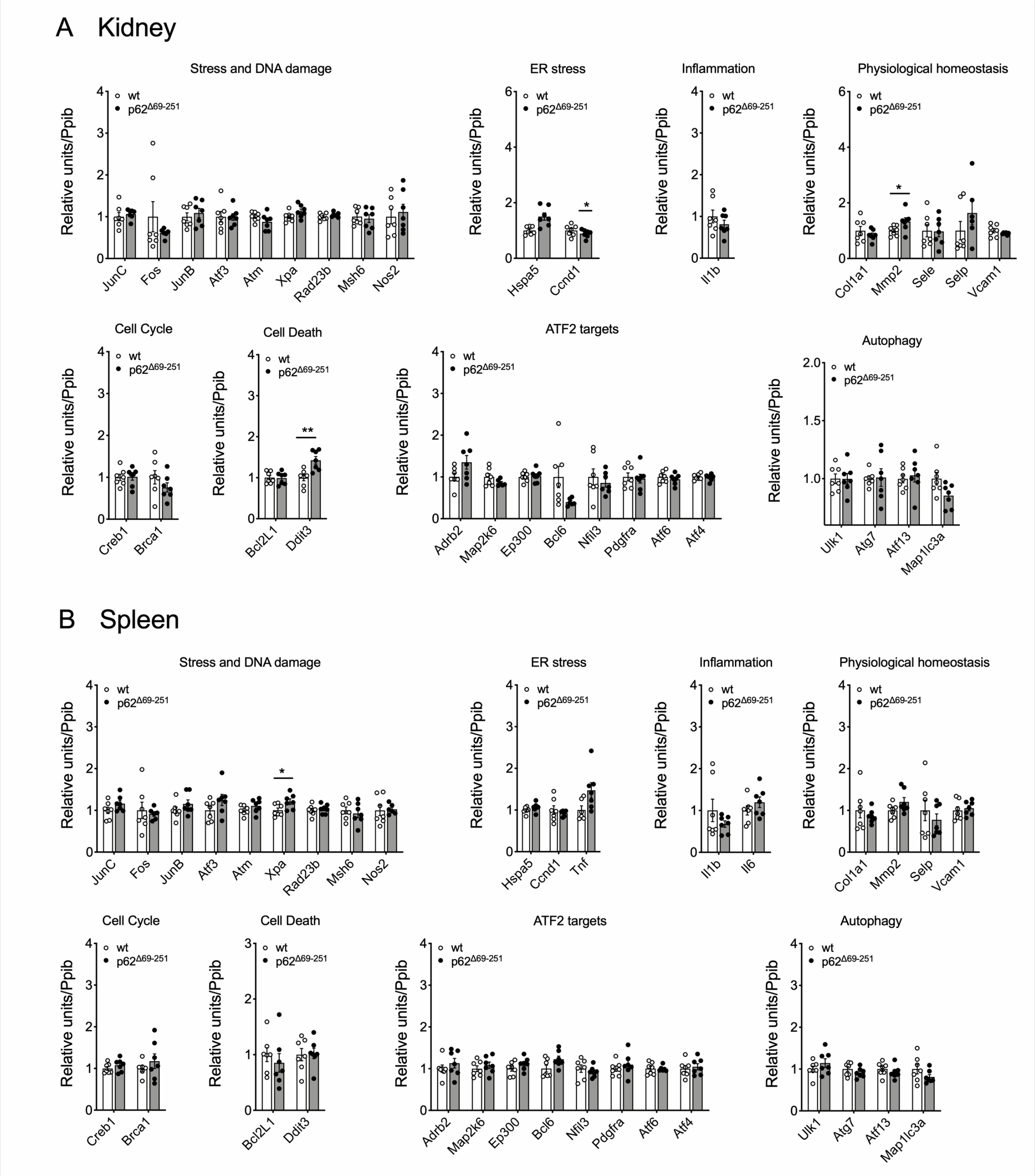

Supplementary Figure 10. Gene expression analysis of kidney and spleen from lean wt or p62$^{\Delta 69\text{-}251}$ mice. Analysis of ATF2 target effects on stress and DNA damage, ER stress, inflammation, physiological homeostasis, cell cycle, cell death and autophagy in kidney (A) and spleen (B) of 14 wk old male C57Bl/6J wt or p62$^{\Delta 69\text{-}251}$ mice. Data have been analyzed using 2-sided 2-tailed ttest. Data represent mean ± SEM (n=6-7 mice per group). *$p < 0.05$; **$p < 0.01$; ***$p < 0.001$. The exact p-values are given in the Data Source File. For Abbreviations please see Suppl. Fig. 9.
