# Supplementary material for: The scaffold protein p62 regulates adaptive thermogenesis through ATF2 nuclear target activation
Source: Nat Commun. 2020 May 8;11:2306. doi: 10.1038/s41467-020-16230-8 (PMC7211001; doi:10.1038/s41467-020-16230-8)
Supplement: Supplementary file 2 — Reporting Summary [file 41467_2020_16230_MOESM2_ESM.pdf]

nature research

Corresponding author(s): Timo D. Müller

Last updated by author(s): Mar 9, 2020

Reporting Summary

Nature Research wishes to improve the reproducibility of the work that we publish. This form provides structure for consistency and transparency in reporting. For further information on Nature Research policies, see Authors & Referees and the Editorial Policy Checklist .

Statistics

For all statistical analyses, confirm that the following items are present in the figure legend, table legend, main text, or Methods section.

n/a	Confirmed	
☐	☒	The exact sample size (*n*) for each experimental group/condition, given as a discrete number and unit of measurement
☐	☒	A statement on whether measurements were taken from distinct samples or whether the same sample was measured repeatedly
☐	☒	The statistical test(s) used AND whether they are one- or two-sided<br>*Only common tests should be described solely by name; describe more complex techniques in the Methods section.*
☐	☒	A description of all covariates tested
☐	☒	A description of any assumptions or corrections, such as tests of normality and adjustment for multiple comparisons
☐	☒	A full description of the statistical parameters including central tendency (e.g. means) or other basic estimates (e.g. regression coefficient) AND variation (e.g. standard deviation) or associated estimates of uncertainty (e.g. confidence intervals)
☐	☒	For null hypothesis testing, the test statistic (e.g. *F*, *t*, *r*) with confidence intervals, effect sizes, degrees of freedom and *P* value noted<br>*Give P values as exact values whenever suitable.*
☒	☐	For Bayesian analysis, information on the choice of priors and Markov chain Monte Carlo settings
☒	☐	For hierarchical and complex designs, identification of the appropriate level for tests and full reporting of outcomes
☐	☒	Estimates of effect sizes (e.g. Cohen's *d*, Pearson's *r*), indicating how they were calculated

Our web collection on statistics for biologists contains articles on many of the points above.

Software and code

Policy information about availability of computer code

Data collection	No custom made code or algorithm was used in the manuscript.
Data analysis	no custom made code or algorithm used in the manuscript. Statistical analysis were made using GraphPad Prism (Version 8.3.0). Quantification of western blots was done using Image Studio lite v5.2. Analysis of energy expenditure was done using ANCOVA with body weight as covariate using SPSS (Version 24).

For manuscripts utilizing custom algorithms or software that are central to the research but not yet described in published literature, software must be made available to editors/reviewers. We strongly encourage code deposition in a community repository (e.g. GitHub). See the Nature Research guidelines for submitting code & software for further information.

Data

Policy information about availability of data

All manuscripts must include a data availability statement. This statement should provide the following information, where applicable:

- Accession codes, unique identifiers, or web links for publicly available datasets
- A list of figures that have associated raw data
- A description of any restrictions on data availability

A data availability statement is given in the statistics section: The data used for the statistical analysis are provided in the data source file or are available from the corresponding author upon reasonable request. Also the GraphPad Prizm-derived report on the statistical analysis is provided along with the raw data for each study in the data source file. This analysis report contains the mean difference between the treatment groups for each time point, the 95% confidence intervals for each time point, the significance summary for each time point and the exact p-value for each time point (if given by the statistical software implemented in GraphPadPrizm V.8.3.0. Notably, in case of significance with p<0.0001, GraphPad Prizm V.8.3.0 does not give exact p-values and rather states that significance is p<0.0001 (see data source files for examples).

Field-specific reporting

Please select the one below that is the best fit for your research. If you are not sure, read the appropriate sections before making your selection.

☒ Life sciences ☐ Behavioural & social sciences ☐ Ecological, evolutionary & environmental sciences

For a reference copy of the document with all sections, see nature.com/documents/nr-reporting-summary-flat.pdf

Life sciences study design

All studies must disclose on these points even when the disclosure is negative.

Sample size	For animal studies, sample sizes were calculated based on a power analysis assuming that a greater or equal (>/=) 5 g difference in body weight between genotypes can be assessed with a power of >/= 75% when using a 2-sided statistical test under the assumption of a standard deviation of 3.5 and an alpha level of 0.05.
Data exclusions	no data were excluded from the analysis unless Grubbs test for outlier identified a sign. outlier that justified its exclusion. Outliers are stated in the data source file.
Replication	key findings were independently confirmed in three different p62 mutant mouse models. In vitro data have been replicated independently as indicated in the figure legends. Statements of successful replication of in vitro data are indicated in the figure legends
Randomization	Animals were assigned to groups based on their genotype (WT or KO). At study start, only age-matched mice were included in the studies. There were no other covariats controlled.
Blinding	Investigators were not blinded to genotypes and treatment conditions since all people performing animal studies by law need to be able at any time to show federal animal protocol approval, study designs, results, treatments as well as number and genotypes of used animals to federal authorities upon spontaneous inspections by the governmental authorities.

Reporting for specific materials, systems and methods

We require information from authors about some types of materials, experimental systems and methods used in many studies. Here, indicate whether each material, system or method listed is relevant to your study. If you are not sure if a list item applies to your research, read the appropriate section before selecting a response.

Materials & experimental systems

n/a	Involved in the study	
☐	☒	Antibodies
☐	☒	Eukaryotic cell lines
☒	☐	Palaeontology
☐	☒	Animals and other organisms
☒	☐	Human research participants
☒	☐	Clinical data

Methods

n/a	Involved in the study	
☒	☐	ChIP-seq
☒	☐	Flow cytometry
☒	☐	MRI-based neuroimaging

Antibodies

Antibodies used

ATF2 (Bethyl; #A301-649A; Dilution: 1:300)
ATF2 (Cell Signaling; #35031; Dilution: 1:1000)
ATF2 (Santa Cruz; #sc-187; Dilution: 1:500)
ATF2 (Santa Cruz; #sc-242(F2BR1); Dilution 1:70)
p62 (Protein Tech; #18420-1AP; Dilution1:100)
p62 (Abcam; #ab101266; Dilution: 1:500)
GAPDH (Santa Cruz; #365062; Dilution: 1:10000)
GAPDH (Santa Cruz; #sc-32233; Dilution: 1:20000)
H3 (Abcam; #ab1791; Dilution 1:25000)
p-ATF2 (Cell signaling; #9225; Dilution: 1:1000)
p-ATF2 (Thermo Fisher Scientific; #05891; Dilution: 1:2000)
p-p38 (Cell Signaling, #4511; Dilution: 1:800)
p-p38(Cell signaling; #9211; Dilution: 1:400)
p38 (Cell Signaling, #9212; Dilution: 1:1000)
p38alpha (Santa Cruz; #sc-728; Dilution: 1:500)
Ucp1 (Abcam, #ab23841; Dilution: 1:2000)
Ucp1 (Abcam; #ab10983; Dilution: 1:6000)

p-PKC (Cell Signaling, #2060; Dilution: 1:2000)
ERK1/2 (Cell Signaling, #9102; Dilution: 1:2000)
Anti-HA (C29F4) (Cell Signaling, Frankfurt, Germany; # 3724S; Dilution: 1:1000)
Anti-FLAG-M2 (Sigma Aldrich, Munich, Germany; # F1804; Dilution: 1:2000)
Anti-beta-actin (Cell Signaling, Frankfurt, Germany; # 4967S; Dilution: 1:2000)
Anti-rabbit (Sigma Aldrich, Munich, Germany; # A3687; Dilution: 1:20,000)
Anti-mouse (Sigma Aldrich, Munich, Germany; #A3562; Dilution: 1:20,000)
LC3A/B (Cell Signaling, #4108; Dilution: 1:2000)
ATG7 (Cell Signaling, #8558; Dilution: 1:2000)
alpha Tubulin (Santa Cruz; #sc-8035; Dilution: 1:1000)

Validation

The Bethyl #A301-649A antibody is recommended by the manufacturer for immunoprecipitation (IP) analysis in human and mouse samples. Validation according to the manufacturer using western blot followed by IP in whole cell lysate (1 mg for IP, 20% of IP loaded) from HeLa cells. https://www.bethyl.com/product/A301-649A and https://www.bethyl.com/product/pdf/A301-649A.pdf.

The Cell Signaling; #35031 monoclonal antibody is recommended by the manufacturer for detection of ATF2 in human, mouse and ra samplest. Validation according to the manufacturers website using western blot analysis of extracts from HeLa cells, mock transfected (-) or transfected with SignalSilence® ATF-2 siRNA #6443 (+), using ATF-2 (D4L2X) XP® Rabbit mAb or β-Actin (D6A8) Rabbit mAb #8457 https://www.cellsignal.de/products/primary-antibodies/atf-2-d4l2x-xp-rabbit-mab/35031. Product citations include Yi et al., Cell Rep. 2019;29:2621-2633.e4; Kim et al., BMC Complement Altern Med. 2019;19:291. doi: 10.1186/s12906-019-2720-4 and Fearnley et al., Biol Open. 2019 May 17;8(5). pii: bio034215. doi: 10.1242/bio.034215.

The Santa Cruz #sc-187 antibody is recommended by the manufacturer for western blot, ChIP and immunohistochemistry in domestic dog, human, mouse, rat samples. Validation according to the manufacturers website using western blot analysis in K-562 nuclear extracts and in human primary fibroblasts. Use of this antibody is reported in 163 publications, including Rajbhandari et al., Cell 2018;172,1-2:218-233.e17; Salinas-Abarca et al., Molecular Pain 2018;14:1-14 DOI: 10.1177/1744806918787427 and Howe et al., 2017;12:e0185619. https://www.citeab.com/antibodies/805945-sc-187-atf-2-antibody-c-19

The Santa Cruz #sc-242(F2BR1) antibody is recommended by the manufacturer for immunofluorescence, immunoprecipitation, and western blot analysis in human, mouse and rat samples. Validation according to the manufacturers website using western blot analysis of in Hep G2 whole cell lysate and western blot analysis of ATF-2 expression in K-562 and Jurkat nuclear extracts and MDA-MB-231, MOLT-4 and HL-60 whole cell lysates https://www.scbt.com/p/atf-2-antibody-f2br-1. The use of this antibody has been reported in 62 publications, including Liu et al., PlosOne 2013; https://doi.org/10.1371/journal.pone.0078253; Hassan et al. 2005, Virology 333;324-336 and Akagawa et al., Biochemical and Biophysical Research Communications 2003;300:600-608, https://doi.org/10.1016/S0006-291X(02)02890-5

The Protein Tech #18420-1AP antibody is recommended by the manufacturer for immunofluorescence, immunoprecipitation, and western blot analysis in human, mouse and rat samples. Validation of western blot applications according to the manufacturer website in HEK-293 cells subjected to SDS PAGE followed by western blot with 18420-1-AP(SQSTM1 antibody) at dilution of 1:1000 incubated at room temperature for 1.5h. Antibody specificity was also validated using an si-p62 vs si-control mediated knock-down of p62 in HEK293 cells https://www.ptglab.com/Products/SQSTM1-Antibody-18420-1-AP.htm. The use of this antibody has been reported in 305 publications, including Su et al., Toxicology. 2016 Jul 1;363-364:48-57. doi: 10.1016/j.tox.2016.07.002, Hou et al., Sci Rep. 2019 Jul 31;9(1):11087. doi: 10.1038/s41598-019-47597-4 and Xiao et al., Cell Death Dis. 2018 Feb 7;9(2):160. doi: 10.1038/s41419-017-0228-8.

The Santa Cruz #365062 antibody is recommended by the manufacturer for immunofluorescence, immunoprecipitation, and western blot analysis in human, mouse and rat samples. Validation of western blot applications according to the manufacturer website using Simultaneous direct near-infrared western blot analysis of GAPDH expression, detected with GAPDH (G-9) Alexa Fluor® 680: sc-365062 AF680 and β-Actin expression, detected with β-Actin (C4) Alexa Fluor® 790: sc-47778 AF790 in Hep G2, HeLa, A549, RAW 264.7 and KNRK whole cell lysates. Blocked with UltraCruz® Blocking Reagent: sc-516214. https://datasheets.scbt.com/sc-365062.pdf. Selected product citations include Tang et al. 2006, Anticancer Drugs 17: 297-305, Saba et al. 2019, Mol Cell Neurosci. 94: 41-51 and Ito et al. 2019. Pharm. Res. 36: 39.
The Santa Cruz; #sc-32233 antibody is recommended by the manufacturer for western blot, immunoprecipitation and immunofluorescence in human samples. Validation according to the manufacturer website using western blot analysis of GAPDH expression in non-transfected 293T: sc-117752, human GAPDH transfected 293T: sc-113887 and KNRK whole cell lysates. https://www.scbt.com/de/p/gapdh-antibody-6c5. Selected product citations include Marrocco et al. 2019. J. Mol. Cell. Cardiol. 128: 212-226, Kim et al., 2019. Nat Commun 10(1):3991. doi: 10.1038/s41467-019-11867-6 and Wu et al., 2019, Cell Death Dis. 10(2):37. doi: 10.1038/s41419-018-1048-1.

The Abcam #ab1791 antibody is recommended by the manufacturer for western blot and hromatin immunoprecipitation in mouse, rat, chicken, dog and human samples. Validation according to the manufacturer using western blot in HeLa, Drosophila embryo nuclear extract, NIH/3T3, S.cerevisiae (Y190) and S.pombe whole cell lysates. ICC/IF: Methanol fixed HeLa cells. ChIP: Chromatin from HeLa cells. IHC-Fr: Mouse brain tissue https://www.abcam.com/histone-h3-antibody-nuclear-loading-control-and-chip-grade-ab1791.html. The use of this antibody has been reported in 2898 publications, including Cassidy et al., 2020; Nat Commun 11:307; Abu-Zhayia et al., 2019; J Mol Cell Biol 11:804-806 and Xu et al.,2019; Mol Med Rep 19:4249-4255.

The Cell signaling #9211 is recommended by the manufacturer for western blot, immunoprecipitation and immunofluorescence in mouse, rat and human samples. Validation according to the manufacturer website using western blot analysis of extracts from C6 cells, untreated or anisomycin-treated, and NIH/3T3 cells, untreated or UV-treated, using Phospho-p38 MAPK (Thr180/

Tyr182) Antibody or p38 MAPK Antibody #9212 https://www.cellsignal.de/products/primary-antibodies/phospho-p38-mapk-thr180-tyr182-antibody/9211. The use of this antibody has been reported in 1682 publications, including Li et al., Am J Transl Res. 2019 Dec 15;11(12):7410-7421; Park et al., Sci Rep. 2019;9(1):19950 and Heinonen et al., Front Immunol. 2019 6;10:2713.

The Cell signaling #9225 antibody is recommended by the manufacturer for western blot and immunohistochemistry in human, mouse, rat and monkey samples. Validation according to the manufacturer using western blot analysis of extracts from 293 cells, untreated or UV-treated, NIH/3T3 cells, untreated or anisomycin-treated, and C6 cells, untreated or anisomycin-treated, using Phospho-ATF-2 (Thr69/71) Antibody. https://media.cellsignal.com/pdf/9225.pdf#_ga=2.194431725.1584204082.1583497501-340760286.1583497501. The use of this antibody has been reported in 45 publications, including Abdel-Hafiz, H.A. et al. (1992) Mol. Endocrinol. 6, 2079–2089, Gupta, S. et al. (1995) Science 267, 389–393, van Dam, H. et al. (1995) EMBO J. 14, 1798–1811 and Livingstone, C. et al. (1995) EMBO J. 14, 1785–1797.

The Thermo Fisher Scientific #05891 antibody is recommended by the manufacturer for western blot, immunoprecipitation in human, mouse and rat samples. Validation and product quality is according to the manufacturer routinely evaluated by immunoblot with RIPA lysates from anisomycin treated 3T3/NIH cells and has been demonstrated by using western blot analysis of untreated or anisomycin treated 3T3/NIH cell lysates probed with anti-phsopho-ATF2 (Thr69/71), clone AW65 (0.2µg/ml). Selected product citations include Salameh et al., J Biol Chem. 2010 Jul 23;285(30):23096-104 and Delhase et al., Proc Natl Acad Sci U S A. 2012 Jan 24;109(4):E177-86. https://www.fishersci.com/shop/products/anti-phospho-atf2-thr69-71-a/05891mi

The Cell Signaling #4511antibody is recommended by the manufacturer for western blot, immunoprecipitation and other applications in human, mouse, rat and monkey samples. Validation according to the manufacturer website in western blot analysis of extracts from COS and 293 cells, untreated or UV-treated, using Phospho-p38 MAPK (Thr180/Tyr182) (D3F9) XP® Rabbit mAb or p38 MAPK Antibody #9212. https://www.cellsignal.de/products/primary-antibodies/phospho-p38-mapk-thr180-tyr182-d3f9-xp-rabbit-mab/4511. The use of this antibody has been reported in 1196 publications, including Bi et al., Oxid Med Cell Longev. 2020 Jan 2;2020:6946037, Ding et al., J Neuroinflammation. 2020 Jan 13;17(1):19 and Wang et al., Theranostics. 2020 Jan 1;10(1):231-246

The Cell Signaling #9212 antibody is recommended by the manufacturer for western blot, immunohistochemistry and other applications in human, mouse, rat and monkey samples. Validation for western blot analysis according to the manufacturer website using extracts from HeLa cells, transfected with 100 nM SignalSilence® Control siRNA (Unconjugated) #6568 (-), SignalSilence® p38 MAPK siRNA I #6564 (+) or SignalSilence® p38 MAPK siRNA II (+), using p38 MAPK Antibody #9212 and α-Tubulin (11H10) Rabbit mAb #2125. The p38 MAPK antibody confirms silencing of p38 MAPK expression while the α-Tubulin (11H10) Rabbit mAb is used to control for loading and specificity of p38 MAPK siRNA. https://www.cellsignal.de/products/primary-antibodies/p38-mapk-antibody/9212. The use of this antibody has been reported in 2088 publications, includingLi et al., Int J Clin Exp Pathol. 2019 Feb 1;12(2):477-487; Barata and Dick, Redox Biol. 2020 Jan;28:101340. doi: 10.1016/j.redox.2019.101340 and Park and Helfman, Sci Rep. 2019 Dec 27;9(1):19950. doi: 10.1038/s41598-019-56276-3.

The Santa Cruz #sc-728 antibody is recommended by the manufacturer for western blot and ELIA in immunoprecipitation and immunofluorescence in mouse, rat and human samples. Validation according to the manufacturer using Western blot analysis of p38 expression in Jurkat, KNRK , A-431 and MCF7 whole cell lysates. Antibodies tested include p38 (N-20): sc-728 and p38 (C-20): sc-535. https://search.cosmobio.co.jp/cosmo_search_p/search_gate2/docs/SCB_/SC728.20070822.pdf. The use of this antibody has been reported in 124 publications, including Yang et al., Mol Med Rep. 2018 Jul;18(1):973-980. doi: 10.3892/mmr.2018.9024; Nernpermpisooth et al., Exp Ther Med. 2017 Dec;14(6):5793-5800. doi: 10.3892/etm.2017.5272 and Chen et al., PLoS One. 2017 Oct 24;12(10):e0186780. doi: 10.1371/journal.pone.0186780.

The Abcam #ab101266 antibody is recommended by the manufacturer for western blot, immunoprecipitation and immunhistochemistry in mouse and human samples. Validation according to the manufacturer's website by detection of Human SQSTM1 / p62 By Immunoprecipitation in 1mg whole HeLa cells lysate (20% of IP loaded) using ab101266 at 3µg/mg of lysate. Subsequent WB detection was performed using ab101266 at 0.4µg/ml. https://www.abcam.com/sqstm1-p62-antibody-ab101266.html#description_images_1. The use of this antibody has been reported in 7 publications, including Yang et al., Autophagy. 2019 Jul 18:1-20. doi: 10.1080/15548627.2019.1643184; Zhuge et al., Front Immunol. 2018 Apr 17;9:788. doi: 10.3389/fimmu.2018.00788 and Shen et al., Redox Biol. 2018 Sep;18:138-157. doi: 10.1016/j.redox.2018.07.004.

The Abcam #ab23841 antibody is recommended by the manufacturer for western blot and immunohistochemistry in mouse, rat and dog samples. Validation according to the manufacturer website using western blot analysis of Anti-UCP1 antibody (ab23841) at 1 µg/ml in Adult Mouse Brown Adipose Tissue Lysate and Adult Rat Brown Adipose Tissue Lysate with Lysates/proteins at 20 µg per lane. https://www.abcam.com/ucp1-antibody-ab23841.html#description_images_1. The use of this antibody has been reported in 83 publications, including Dimitriadis et al., Cytokine. 2019 Jan;113:248-255. doi: 10.1016/j.cyto.2018.07.013, Sepa-Kishi et al., Am J Physiol Cell Physiol. 2019 Mar 1;316(3):C365-C376. doi: 10.1152/ajpcell.00122.2018 and Cao et al., Physiol Rep. 2019 Mar;7(6):e14031. doi: 10.14814/phy2.14031.

.

The Abcam #ab10983 antibody is recommended by the manufacturer for western blot and immunhistochemistry in mouse, rat and spermophilius tridecemlineatus samples. Validation according to the manufacturer website using western blot analysis in mouse, rat and spermophilius tridecemlineatus. The antibody Detects a band of approximately 32 kDa when using a minimum dilution of 1/1000 in extract of rat brown adipose tissue (BAT) mitochondria or an extract of E.coli expressing recombinant mouse UCP1. Additional weak bands may be detected in some preparations of BAT extracts. Staining of the UCP1 band is specifically inhibited with the immunizing peptide. https://www.abcam.com/ucp1-antibody-ab10983.html. The antibody has been used in 368 publications, including Fujimoto et al., J Cell Biochem. 2019 Jan;120(1):821-835. doi: 10.1002/jcb.27443, Cunarro et al., Mol Nutr Food Res. 2019 Jan;63(2):e1801096. doi: 10.1002/mnfr.201801096 and Nishikawa et al., J Agric Food Chem. 2019 Feb 20;67(7):1948-1954. doi: 10.1021/acs.jafc.8b06647.

The Cell Signaling #2060 antibody is recommended by the manufacturer for western blot analysis in mouse, rat, human and

monkey samples. Validation according to the manufacturer website using western blot analysis of baculovirus-expressed PKC isoforms, using Phospho-PKC (pan) (zeta Thr410) (190D10) Rabbit mAb. The use of this antibody is reported in 36 publications, including Yuang et al., Mol Med Rep. 2019 Aug;20(2):1093-1102. doi: 10.3892/mmr.2019.10330, Liang et al., BMC Ophthalmol. 2019 Mar 18;19(1):79. doi: 10.1186/s12886-019-1087-0 and Zhang et al., Oncogene. 2019 Feb;38(7):1121-1135. doi: 10.1038/s41388-018-0498-3.

The Cell Signaling #9102 antibody is recommended by the manufacturer for western blot, immunoprecipitation and immunhistochemistry in in mouse, rat and human samples. Validation according to the manufacturer website using western blot analysis of extracts from HeLa cells transfected with 100 nM control siRNA #6201 (-) or p44 MAPK (Erk1) siRNA (+), using p44/42 MAPK (Erk1/2) Antibody #9102 and GCK Antibody #3782. The Erk1/2 antibody confirms silencing of Erk1 expression, and GCK Antibody is used to control for loading and specificity of p44 MAPK (Erk1) siRNA. https://en.cellsignal.de/products/primary-antibodies/p44-42-mapk-erk1-2-antibody/9102. The use of this antibody is reported in 3970 publications, including Lin et al., Int J Obes (Lond). 2020 Feb;44(2):466-474. doi: 10.1038/s41366-019-0379-z, Hamyeh et al., Theranostics. 2020 Jan 1;10(3):1016-1032. doi: 10.7150/thno.38537 and Liu et al., Front Immunol. 2019 Dec 20;10:2952. doi: 10.3389/fimmu.2019.02952.

The Cell Signaling #4108 antibody is recommended by the manufacturer for western blot and immunoprecipitation in mouse, rat and human samples. Validation according to the manufacturer website using western blot analysis of extracts from various cell lines, untreated or treated with chloroquine (50 µM, overnight) using LC3A/B Antibody. https://en.cellsignal.de/products/primary-antibodies/lc3a-b-antibody/4108. The use of this antibody is reported in 377 publications, including Wang et al., J Cell Physiol. 2020 Mar;235(3):2722-2737. doi: 10.1002/jcp.29176, Das et al., Cell Death Dis. 2020 Jan 23;11(1):50. doi: 10.1038/s41419-020-2249-y and Huang et al., Oxid Med Cell Longev. 2019 Nov 25;2019:1305049. doi: 10.1155/2019/1305049.

The Cell Signaling #8558 antibody is recommended by the manufacturer for western blot and immunoprecipitation in mouse, rat and human samples. Validation according to the manufacturer website using western blot analysis of extracts from various cell lines using Atg7 (D12B11) Rabbit mAb and using western blot analysis of extracts from HeLa cells, transfected with 100 nM SignalSilence® Control siRNA (Unconjugated) #6568 (-) or SignalSilence® Atg7 siRNA I #6604 (+), using Atg7 (D12B11) Rabbit mAb or α-Tubulin (11H10) Rabbit mAb #2125. The Atg7 (D12B11) Rabbit mAb confirms silencing of Atg7 expression, while the α-Tubulin (11H10) Rabbit mAb is used as a loading control. https://en.cellsignal.de/products/primary-antibodies/atg7-d12b11-rabbit-mab/8558. The use of this antibody is reported in 219 publications, including Yeon et al., Front Oncol. 2019 Nov 14;9:1240. doi: 10.3389/fonc.2019.01240, Minocha et al., Stem Cell Res Ther. 2019 Dec 4;10(1):370. doi: 10.1186/s13287-019-1476-6 and Asrani et al., J Clin Invest. 2019 Dec 2;129(12):5584-5599. doi: 10.1172/JCI128287.

The Santa Cruz #sc-8035 antibody is recommended by the manufacturer for western blot and immunoprecipitation in mouse, rat and human. Validation according to the manufacturer website using western blot analysis of α Tubulin expression in NIH/3T3, C2C12, NAMALWA, A-673, PC-12 and C6 whole cell lysates. Selected publications using this antibody include Zhang, L., et al. 2000. Role of BAX in the apoptotic response to anticancer agents. Science 290: 989-992, Babagana, M., et al. 2017. P21-activated kinase 1 regulates resistance to BRAF inhibition in human cancer cells. Mol. Carcinog. 56: 1515-1525 and Laporte, A.N., et al. 2017. HDAC and proteasome inhibitors synergize to activate pro-apoptotic factors in synovial sarcoma. Plos One 12: e0169407. https://datasheets.scbt.com/sc-8035.pdf

The Cell Signaling #3724S antibody is recommended by the manufacturer for western blot, immunoprecipitation and ChIP in all species. Validation according to the manufacturer website using western blot analysis of extracts from HeLa cells, untransfected or transfected with either HA-FoxO4 or HA-Akt3, using HA-Tag (C29F4) Rabbit mAb.https://www.cellsignal.com/products/primary-antibodies/ha-tag-c29f4-rabbit-mab/3724. The use of this antibody is reported in 74 publications, including Larabee et al., Sci Rep. 2018 Nov 16;8(1):16931, Valcarcel-Jimenez et al., Cell Death Dis. 2018 Oct 11;9(10):1041 and Hauser et al.,Cell Rep. 2018 Aug 7;24(6):1512-152. https://www.biocompare.com/9776-Antibodies/645410-HATag-C29F4-Rabbit-mAb/#citations

The Sigma Aldrich antibody # F1804 is recommended by the manufacturer for western blot, immunoprecipitation in all species. Validation according to the manufacturer website by demonstration of a single band of protein on a Western Blot from mammalian crude cell lysates by chemiluminescent probing Sensitivity Test. https://www.sigmaaldrich.com/Graphics/COfAInfo/SigmaSAPQM/SPEC/F1/F1804/F1804-BULK_________SIGMA_____.pdf. The use of this antibody has been reported in 4030 publications, including Croft et al., BMC Mol Biol. 2017 May 15;18(1):13, Liu et al., Cell Death Differ. 2019 Sep;26(9):1735-1749 and Amaya et al., Mol Biol Cell. 2019 Aug 15;30(18):2377-2398.

The Cell Signaling #4967S antibody is recommended by the manufacturer for western blot analysis in samples from human, mouse, monkey and other species. Validation according to the manufacturer website by western blot analysis of extracts from HeLa, C2C12, C6, COS, MvLu cells and guinea pig neutrophils (GPN), using β-Actin Antibody. https://www.cellsignal.de/products/primary-antibodies/b-actin-antibody/4967. The use of this antibody has been reported in 1421 publications, including Tian et al., Exp Ther Med. 2020 Mar;19(3):1940-1946, Kong et al., Onco Targets Ther. 2020 Feb 5;13:1099-1108 and Hou et al., Onco Targets Ther. 2020 Jan 31;13:959-973.

The Sigma Aldrich antibody # A3687 is recommended by the manufacturer for western blot and immunhistochemistry. Validation according to the manufacturer website by western blot analysis and detection of Rabbit IgG using 10 µg protein under reducing conditions on an SDS-PAGE gradient (4-20%) gel. The protein was transferred to nitrocellulose, blocked with 5% BSA in 0.05 M Tris and then incubated with the conjugate.https://www.sigmaaldrich.com/content/dam/sigma-aldrich/docs/Sigma/Datasheet/6/a3687dat.pdf. The use of this antibody has been reported in 167 publications, including Manders et al., Br J Cancer. 2002 Sep 23;87(7):772-8, DeGooyer et al., Invest Ophthalmol Vis Sci. 2006 Dec;47(12):5553-60 and Cibelli et al., Eur J Neurosci. 2001 Apr;13(7):1339-48.

The Sigma Aldrich antibody # A3562 is recommended by the manufacturer for western blot and immunhistochemistry in mouse samples. Validation according to the manufacturer website by detection of Surfactant Protein A in bronchoalveolar fluid using alkaline phosphatase conjugated goat anti-mouse IgG as the secondary at µg/ml in TBS/Tween containing final concentration of

0.5M NaCl. https://www.sigmaaldrich.com/catalog/product/sigma/a3562?lang=de&region=DE. The use of this antibody has been reported in 136 publications, including Alam et al., J Virol. 2008 Jan;82(1):115-25, Shao et al., Mol Cell Biol. 2008 Sep;28 (17):5196-208 and Ey et al., Nature. 1979 Oct 11;281(5731):492-3.

Eukaryotic cell lines

Policy information about cell lines

Cell line source(s)	HEK293T cells (ATCC, USA), HEK293FT cells (ATCC, USA)
Authentication	Authentication according to the manufacturer's website:<br>The 293T cell line, originally referred as 293tsA1609neo, is a highly transfectable derivative of human embryonic kidney 293 cells, and contains the SV40 T-antigen.This cell line is competent to replicate vectors carrying the SV40 region of replication. It gives high titers when used to produce retroviruses. It has been widely used for retroviral production, gene expression and protein production. Product related references include DuBridge et al., Mol Cell Biol. 1987 Jan;7(1):379-87 and Pear et al., Proc Natl Acad Sci U S A. 1993 Sep 15;90(18):8392-6. https://www.lgcstandards-atcc.org/Products/All/CRL-3216.aspx?geo_country=de#generalinformation<br><br>The HEK293FT cell line is a hypotriploid human cell line. The modal chromosome number was 64, occurring in 30% of cells. The rate of cells with higher ploidies was 4.2 %. The der(1)t(1;15) (q42;q13), der(19)t(3;19) (q12;q13), der(12)t(8;12) (q22;p13), and four other marker chromosomes were common to most cells. Five other markers occurred in some cells only. The marker der(1) and M8 (or Xq+) were often paired. There were four copies of N17 and N22. Noticeably in addition to three copies of X chromosomes, there were paired Xq+, and a single Xp+ in most cells. Product related references include Da Costa et al., Proc Natl Acad Sci U S A. 1996 Apr 30;93(9):4192-6, Graham et al., J Gen Virol. 1977 Jul;36(1):59-74 and Graham et al., Virology. 1978 May 1;86(1):10-21.https://www.lgcstandards-atcc.org/products/all/CRL-1573.aspx?geo_country=de#generalinformation
Mycoplasma contamination	cell lines were free of mycoplasm contaminations
Commonly misidentified lines<br>(See ICLAC register)	no misidentified cell lines were used in the manuscript

Animals and other organisms

Policy information about studies involving animals; ARRIVE guidelines recommended for reporting animal research

Laboratory animals

Fig. 1 A-C: 31 week old male C57Bl/6J p62 wt or p62delta69-251 mice.
Fig. 1D,E: 24 week old male C57Bl/6J wt or p62delta69-251 mice.
Fig. 1F, 14, 16, 20 week old male C57Bl/6J wt or p62delta69-251 mice.
Fig. 1G 14 week old male C57Bl6/J wt or p62delta69-251 mice.
Fig. 1H 14-30 week old male C57Bl/6J wt or p62delta69-251 mice.
Fig. 1I,J: 30 week old male C57Bl/6J male wt or p62delta69-251 mice.
Fig. 2A: 32 week old male C57Bl/6J wt or p62delta69-251 mice.
Fig. 2B,C: 26 week old male C57Bl/6J wt or p62delta69-251 mice.
Fig. 2D: 26 week old male C57Bl/6J wt or p62delta69-251 mice.
Fig. E: 32 week old male C57Bl/6J wt or p62delta69-251 mice.
Fig. 2F-I: 33 week old male C57Bl/6J wt or p62delta69-251 mice.
Fig. 3A: 7day old male C57Bl/6J wt or p62delta69-251 mice.
Fig. 3B-E: 12 week old male C57Bl/6J wt or p62delta69-251 mice.
Fig. 4A 8 week old male C57Bl/6J Ucp1 cre negative p62flx/flx or Ucp1 cre positive p62flx/flx mice.
Fig. 4B,C: female and male 7 day old male C57Bl/6J Ucp1 cre negative p62flx/flx or Ucp1 cre positive p62flx/flx mice.
Fig. 4D-F: 8-26 week old male C57Bl/6J Ucp1 cre negative p62flx/flx or Ucp1 cre positive p62flx/flx mice.
Fig. 4G-J 6-8 week old male C57Bl/6J Ucp1 cre negative p62flx/flx or Ucp1 cre positive p62flx/flx mice.
Fig. 5A-D: 6-8 week old male C57Bl/6J wt or p62delta69-251 mice.
Fig. 6C,D: 8 week old male C57Bl/6J wt or p62delta69-251 mice.
Fig. 6E: 6-8 week old male C57Bl/6J Ucp1 cre negative p62flx/flx or Ucp1 cre positive p62flx/flx mice.
Fig. 7A: 8 week old male C57Bl/6J wt or p62delta69-251 mice.
Fig. 7B: 17 week old male C57Bl/6J wt or p62-/- mice
Supplementary Fig. 1B: 14 week old male C57Bl/6J wt or p62delta69-251 mice
Supplementary Fig. 1C: 12 week old male C57Bl/6J wt or p62delta69-251 mice
Supplementary Fig. 1D: 32 week old male C57Bl/6J wt or p62delta69-251 mice
Supplementary Fig. 1E: 14 week old male C57Bl/6J wt or p62delta69-251 mice
Supplementary Fig. 1G: 8-10 week old male C57Bl/6J wt or p62delta69-251 mice

Supplementary Fig. 1H: 14 week old male C57Bl/6J wt or p62delta69-251 mice
Supplementary Fig. 2A: 8-30 week old male C57Bl/6J wt or p62delta69-251 mice
Supplementary Fig. 2B: 13-30 week old male C57Bl/6J wt or p62-/- mice
Supplementary Fig. 2C: 13-30 week old male C57Bl/6J wt or aP2 cre positive p62flx/flx mice
Supplementary Fig. 2D: 9-10 week old male C57Bl/6J wt or p62delta69-251 mice
Supplementary Fig. 2E: 13-30 week old male C57Bl/6J wt or p62-/- mice
Supplementary Fig. 2F: 13-30 week old male C57Bl/6J wt or aP2 cre positive p62flx/flx mice
Supplementary Fig. 3A-D: 14 week old male C57Bl/6J wt or p62delta69-251 mice
Supplementary Fig. 4A-F: 14 week old male C57Bl/6J wt or p62delta69-251 mice
Supplementary Fig. 5A-L: 6-8 week old male C57Bl/6J wt or p62delta69-251 mice
Supplementary Fig. 6A-H: 6-8 week old male C57Bl/6J wt or p62delta69-251 mice
Supplementary Fig. 7A-I: 14 week old male C57Bl/6J wt or p62delta69-251 mice
Supplementary Fig. 8A: 25 week old male C57Bl/6J wt or p62-/- mice
Supplementary Fig. 8B: 27 week old male C57Bl/6J Ucp1 cre negative p62flx/flx or Ucp1 cre positive p62flx/flx mice.
Supplementary Fig. 9: 14 week old male C57Bl/6J wt or p62delta69-251 mice
Supplementary Fig. 10: 14 week old male C57Bl/6J wt or p62delta69-251 mice

Wild animals

no field collected animals were used in the study

Field-collected samples

no field collected animals were used in the study

Ethics oversight

Animal experiments were performed in accordance with European guidelines under permission of the local animal ethics committee of the state of Bavaria or Tübingen.

Note that full information on the approval of the study protocol must also be provided in the manuscript.
